# Molecular blueprints for spinal circuit modules controlling locomotor speed in zebrafish

**DOI:** 10.1038/s41593-023-01479-1

**Published:** 2023-11-02

**Authors:** Irene Pallucchi, Maria Bertuzzi, David Madrid, Pierre Fontanel, Shin-ichi Higashijima, Abdeljabbar El Manira

**Affiliations:** 1 Department of Neuroscience, Karolinska Institutet, Stockholm, Sweden; 2 Division of Behavioral Neurobiology, National Institute for Basic Biology, Okazaki, Japan; 3 Neuronal Networks Research Group, Exploratory Research Center on Life and Living Systems (ExCELLS), Okazaki, Japan

**Keywords:** Central pattern generators, Motor neuron

## Abstract

The flexibility of motor actions is ingrained in the diversity of neurons and how they are organized into functional circuit modules, yet our knowledge of the molecular underpinning of motor circuit modularity remains limited. Here we use adult zebrafish to link the molecular diversity of motoneurons (MNs) and the rhythm-generating V2a interneurons (INs) with the modular circuit organization that is responsible for changes in locomotor speed. We show that the molecular diversity of MNs and V2a INs reflects their functional segregation into slow, intermediate or fast subtypes. Furthermore, we reveal shared molecular signatures between V2a INs and MNs of the three speed circuit modules. Overall, by characterizing how the molecular diversity of MNs and V2a INs relates to their function, connectivity and behavior, our study provides important insights not only into the molecular mechanisms for neuronal and circuit diversity for locomotor flexibility but also for charting circuits for motor actions in general.

## Abstract (web summary)

The study by Pallucchi et al. links the molecular identity of motoneuron and V2a interneuron subtypes to their function and uncovers orthogonal transcriptomic rules for their assembly into separate circuit modules controlling locomotor speed.

## Main

A fundamental hallmark of motor actions is the flexibility of the timing, speed and strength that is central to rapid adaptation to the ever-changing world around us. This is particularly apparent during locomotion—a behavior that involves full-body coordination characterized by sudden changes in speed and strength^1–12^. Important insights into the organization of locomotor circuits have been gained by identifying the broadly defined neuronal populations that control distinct features of locomotion (rhythm, pattern, gait)^3,6,7,10,13–17^.

Studies in zebrafish and mice have revealed a modular organization of the locomotor network with dedicated circuits engaged at slow or fast locomotor speeds^5,7,10,15,18–25^. In these circuits, motoneurons (MNs) are the final processing stage that transforms centrally generated locomotor programs into sequences of muscle activity along the body. Whereas the heterogeneity of MNs has long been appreciated, it is only recently that transcriptional profiling has begun to reveal markers for different MN subtypes^26–30^. The flexibility of locomotor movements emerges from the precise organization of the underlying circuit, particularly how excitatory interneurons (INs) drive the different MN subtypes. Electrophysiological analysis in adult zebrafish has revealed that rhythm-generating excitatory V2a INs and MNs that are recruited together are also connected to each other to form three circuit modules engaged in a speed-dependent manner^18,19,23,24,31–33^. However, how their diversity and circuit modularity is imprinted in their molecular makeup remains unclear. Addressing this question will provide key insights into the logic of neuronal diversity and the formation of functional ensembles for flexible behavior.

By leveraging the detailed circuit knowledge in adult zebrafish combined with experimental and genetic accessibility, we assessed the link between the molecular and functional features defining the MN and V2a IN subtypes and their modular circuit organization. We uncover the molecular principles governing the diversity of MNs and V2a INs into three connected functional subtypes. In addition, we reveal the molecular logic that underpins their organization into circuit modules that control locomotor speed.

## Results

### Molecular diversity of MNs

**Extended Data Fig. 1:**
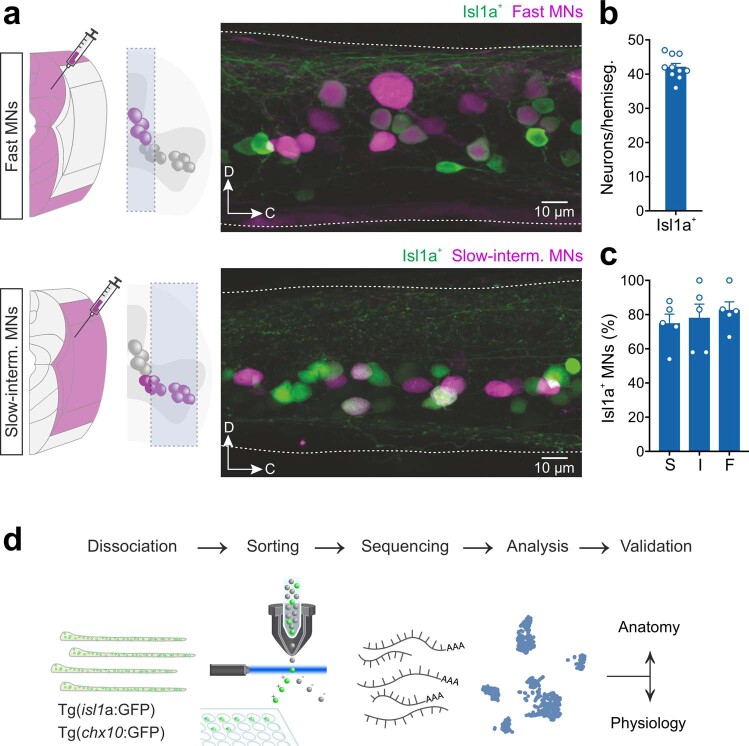
Single-cell RNAseq of GFP^+^ neurons in *isl1a*:GFP and *chx10*:GFP transgenic lines. **a**, Top left: Retrograde dye injection into the fast axial muscles labels dorsomedial fast MNs in the spinal cord. Top right: Lateral view of the medial spinal cord in the transgenic line Tg(*isl1a*:GFP) with fast MNs labeled (magenta). Bottom left: Retrograde dye injection into the slow–intermediate axial muscles labels ventral slow and intermediate MNs. Bottom right: Lateral view of the lateral spinal cord in the transgenic line Tg(*isl1a*:GFP) with slow and intermediate MNs labeled (magenta). **b**, Number of GFP^+^ neurons per hemisegment in the Tg(*isl1a*:GFP) transgenic line (mean ± SEM, *n* = 5 animals). **c**, Percentage of GFP^+^ slow (S), intermediate (I), and fast (F) MNs retrogradely labeled in the transgenic line Tg(*isl1a*:GFP) (mean ± SEM, *n* = 6 animals). **d**, Schematic of the single-cell RNAseq workflow. Source data

**Extended Data Fig. 2:**
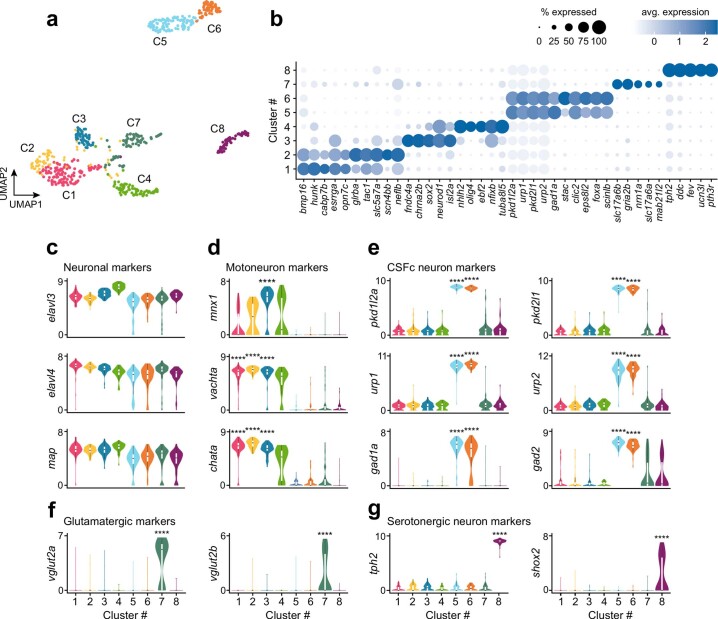
Isl1a^+^ cells cluster analysis. **a**, UMAP of eight color-coded clusters of molecularly defined Isl1a^+^ cells (*n* = 606 cells). **b**, Examples of marker genes selectively expressed in each cluster. **c**, Log-normalized gene expression levels of three neuronal gene markers (differential gene expression analysis, non-parametric Wilcoxon rank sum test with Bonferroni adjusted p-value). **d**, Log-normalized expression levels of MN gene markers enriched in four motoneuronal clusters (C1-4; differential gene expression analysis, non-parametric Wilcoxon rank sum test with Bonferroni adjusted p-value, ****p < 0.0001). **e**, Log-normalized gene expression levels of CSFc neuron markers (differential gene expression analysis, non-parametric Wilcoxon rank sum test with Bonferroni adjusted p-value, ****p < 0.0001). **f**, Log-normalized gene expression levels of two glutamatergic markers (differential gene expression analysis, non-parametric Wilcoxon rank sum test with Bonferroni adjusted p-value, ****p < 0.0001). **g**, Log-normalized gene expression levels of two serotonergic markers (differential gene expression analysis, non-parametric Wilcoxon rank sum test with Bonferroni adjusted p-value, ****p < 0.0001).

The molecular diversity of MNs was examined using single-cell RNA sequencing (scRNA-seq) in 7-week-old zebrafish of the transgenic line Tg(*isl1a*:GFP)^34^. This line captured ~80% of each of the three axial MN subtypes (slow, intermediate and fast; Extended Data Fig. 1a–c). Spinal cords from this line were dissected and dissociated, and green fluorescent protein positive (GFP^+^) cells were collected individually by fluorescence-activated cell sorting (FACS) and sequenced using the Smart-seq2 protocol (Extended Data Fig. 1d)^35,36^. A total of 606 isl1a^+^ neurons passed quality control and were used for cluster analysis. Transcriptomic analysis of isl1a^+^ cells revealed eight distinct clusters, each with selective expression of genetic markers (Extended Data Fig. 2a,b). Whereas all clusters expressed neuronal markers (Extended Data Fig. 2c), only four clusters showed high levels of marker genes for MN identity such as *mnx1*, *slc18a3a* (also known as *vachta*) and *chata* (Extended Data Fig. 2d). Two of the non-MN clusters expressed markers for cerebrospinal-fluid-contacting neurons (Extended Data Fig. 2e). The other two clusters expressed glutamatergic and serotonergic markers, respectively (Extended Data Fig. 2f,g). Only neurons expressing MN markers were used for subsequent analysis.

**Fig. 1:**
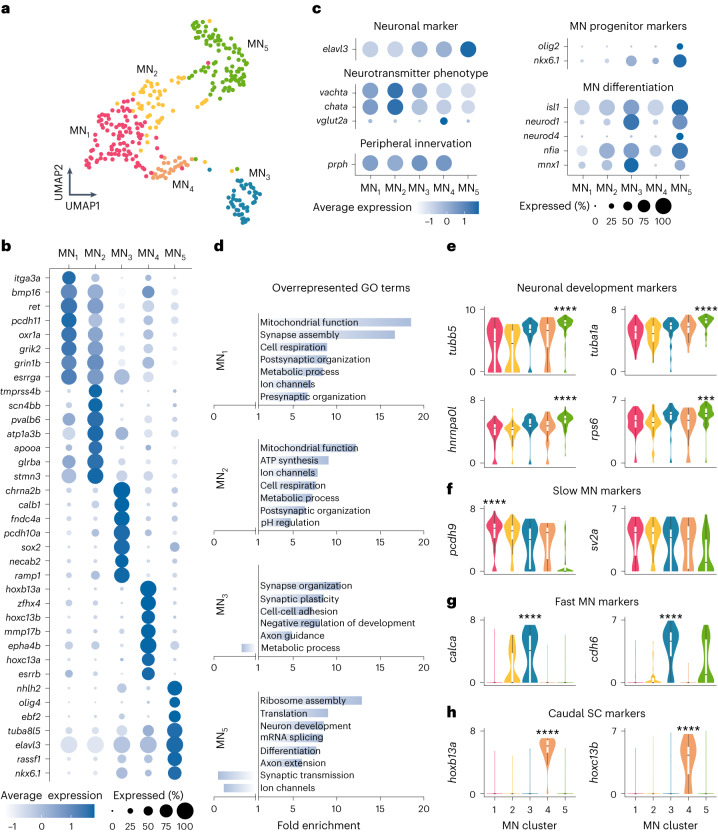
Molecular characterization of MN diversity. **a**, MN single-cell transcriptomes visualized using UMAP of five color-coded, molecularly defined clusters (*n* = 316 cells; MN_1_, *n* = 98; MN_2_, *n* = 51; MN_3_, *n* = 47; MN_4_, *n* = 27; MN_5_, *n* = 93). **b**, Examples of differentially expressed genes in each cluster. The size of the circle reflects the proportion (%) of cells expressing the gene, and the color intensity reflects its average expression level within that cluster. **c**, Normalized expression levels for motoneuronal marker genes. **d**, GO analysis of differentially expressed genes in each cluster. **e**, Log-normalized gene expression levels for neuronal development gene markers (differential gene expression analysis, nonparametric Wilcoxon rank sum test with Bonferroni adjusted *P* value, ***P = 0.0003, ****P < 0.0001). **f**, Log-normalized expression levels of known gene markers of slow MNs in zebrafish (*pcdh9*) or mice (*sv2a*) (differential gene expression analysis, nonparametric Wilcoxon rank sum test with Bonferroni adjusted *P* value, ****P < 0.0001). **g**, Log-normalized expression levels of known gene markers of fast MNs in mice (differential gene expression analysis, nonparametric Wilcoxon rank sum test with Bonferroni adjusted *P* value, ****P < 0.0001). **h**, Log-normalized expression levels for caudal spinal cord (SC) gene markers (differential gene expression analysis, nonparametric Wilcoxon rank sum test with Bonferroni adjusted *P* value, ****P < 0.0001). In **e**–**h**, boxes are bound by 25th and 75th percentiles, center line indicates the median and whiskers extend from minimum to maximum.

The identified MNs were reanalyzed, resulting in five clusters (Fig. 1a,b), of which cluster MN_5_ displayed distinct characteristics of immature neurons. The immature nature of this cluster was supported by the higher expression of markers for MN progenitor (*olig2, nkx6.1*), MN differentiation (*isl1, neurod4, nfia*) and neuronal development markers (Fig. 1c,e). In addition, this cluster lacked the marker for peripheral innervation *prph* and expressed lower levels of motoneuronal transmitter markers (*vachta* and *chata*) (Fig. 1c). This cluster was also enriched with genes related to MN development and less enriched with genes related to electrical and synaptic activity (Fig. 1d).

**Extended Data Fig. 3:**
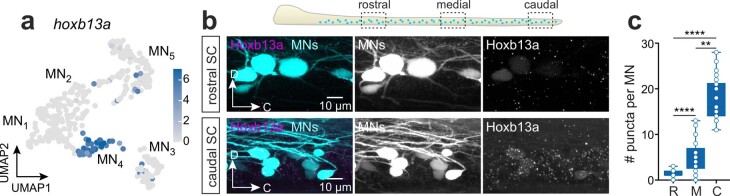
Cluster MN_4_ corresponds to caudal MNs. **a**, UMAP of log-normalized expression of the MN_4_ marker gene *hoxb13a*. **b**, Top: schematic of the analyzed rostrocaudal spinal cord regions. RNAscope *in situ* hybridization of *hoxb13a* in MNs in the rostral and caudal spinal cord regions. **c**, Box and whiskers plot of the number of punta in MNs along the rostral (R), middle (M) and caudal (C) spinal cord. MN_4_ marker gene *hoxb13a* is expressed in a rostrocaudal gradient in the spinal cord (the boxes are bound by the 25^th^ and 75^th^ percentiles, whiskers extend from min. to max.; R: *n* = 17; M: *n* = 21; C: *n* = 22 MNs from 3 animals; one-way ANOVA with Tukey’s post hoc multiple comparisons, **p = 0.0041, ****p < 0.0001). Source data

The remaining four clusters displayed characteristic features of mature MNs, including cholinergic transmitter and peripheral innervation markers (Fig. 1c). Gene ontology (GO) analysis showed that clusters MN_1_ and MN_2_, but not MN_3_, were enriched with genes related to high metabolic activity associated with sustaining firing pattern usually found in slow MNs (Fig. 1d). To determine whether these clusters correspond to functional MN subtypes, we examined their expression of known marker genes of slow and fast MNs. Cluster MN_1_ was differentially enriched with a marker gene of slow MN in zebrafish, *pcdh9* (ref. ^37^), while a marker for slow MNs in mammals, *sv2a*, was not differentially expressed in any of the clusters (Fig. 1f)^38^. In contrast, marker genes of fast MNs in mammals, *calca* and *cdh6*, were selectively enriched in cluster MN_3_ (Fig. 1g)^27,39,40^. On the other hand, cluster MN_4_ was characterized by the selective overexpression of several *hox13* genes (Fig. 1h), which could merely reflect the caudal location of these MNs rather than a specific MN subtype. This was confirmed using RNAscope in situ hybridization analysis, which showed that *hoxb13a* was indeed expressed only in MNs located in the caudal spinal cord (Extended Data Fig. 3a–c). Overall, these results reveal three MN subtypes in adult zebrafish that are defined by marker genes for slow or fast MNs.

### Linking MN molecular diversity and function

**Fig. 2:**
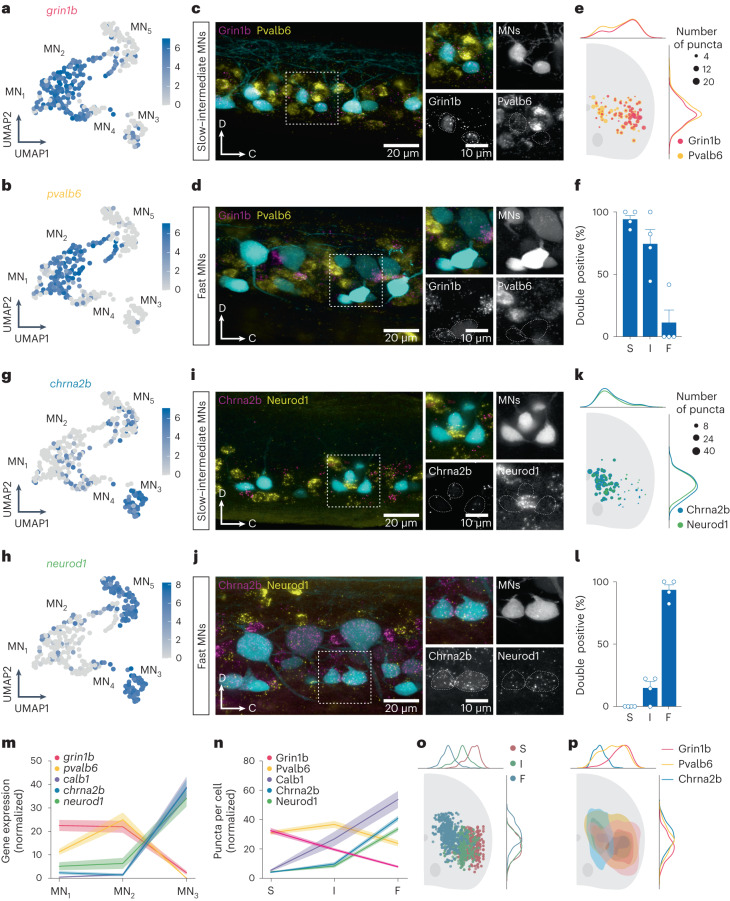
Validation of new molecular markers for MN subtypes. **a**, UMAP of log-normalized expression of *grin1b* in MN clusters. **b**, UMAP of log-normalized expression of *pvalb6* in MN clusters. **c**, Lateral view of a spinal cord segment showing RNAscope in situ hybridization of *grin1b* and *pvalb6* in slow–intermediate MNs. **d**, RNAscope in situ hybridization of *grin1b* and *pvalb6* in fast MNs. **e**, Normalized soma position of Grin1b^+^ and Pvalb6^+^ MNs in the spinal cord, coronal view of one side. The size of the circles reflects the number of puncta per cell (*n* = 4 animals). **f**, Graph showing the percentage of slow (S), intermediate (I) and fast (F) MNs coexpressing *grin1b* and *pvalb6* (mean ± s.e.m., *n* = 4 animals). **g**, UMAP of log-normalized expression of *chrna2b* in MN clusters. **h**, UMAP of log-normalized expression of *neurod1* in MN clusters. **i**, Lateral view of a spinal cord segment showing RNAscope in situ hybridization of *chrna2b* and *neurod1* in slow–intermediate MNs. **j**, RNAscope in situ hybridization of *chrna2b* and *neurod1* in fast MNs. **k**, Normalized soma position of Chrna2b^+^ and Neurod1^+^ MNs in a coronal section of the spinal cord (*n* = 4 animals). **l**, Percentage of S, I and F MNs coexpressing *chrna2b* and *neurod1* (mean ± s.e.m., *n* = 4 animals). **m**, Normalized and averaged expression of *grin1b*, *pvalb6*, *calb1, chrna2b* and *neurod1* in MN clusters (MN_1–3_) (mean ± s.e.m.). **n**, Normalized and averaged number of puncta in Grin1b^+^, Pvalb6^+^, Calb1^+^, Chrna2b^+^ and Neurod1^+^ in S, I and F MNs (mean ± s.e.m.; *n* = 4 animals). **o**, Soma position of S, I and F MNs. **p**, Spatial distribution of Grin1b^+^, Pvalb6^+^ and Chrna2b^+^ MNs. Source data

**Extended Data Fig. 4:**
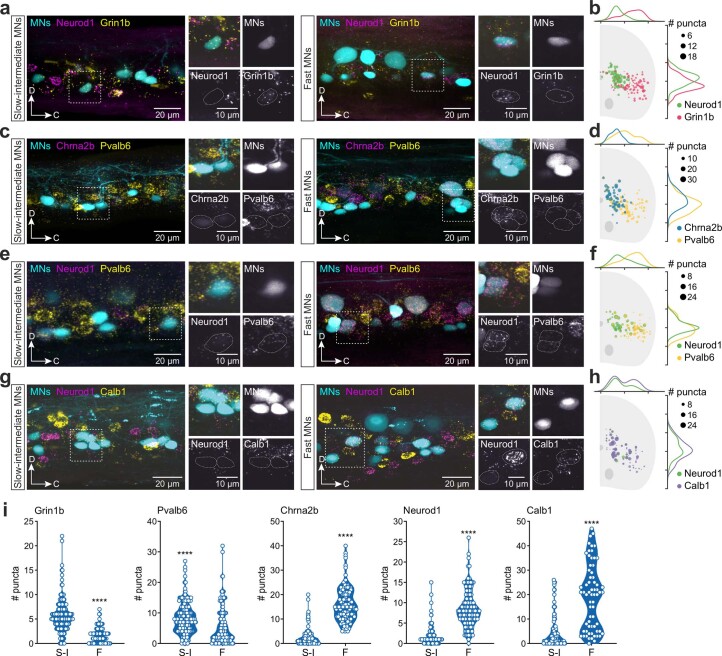
Spatial expression of markers for the different MN clusters. **a**, RNAscope *in situ* hybridization of *grin1b* and *neurod1* in slow–intermediate and fast MNs. **b**, Normalized soma position of Grin1b^*+*^ and Pvalb6^+^ MNs in the spinal cord. The size of the circles reflects the number of puncta per cell (# puncta) (*n* = 5 animals). **c**, RNAscope *in situ* hybridization of *pvalb6* and *chrna2b* in slow–intermediate or fast MNs. **d**, Normalized soma position of Pvalb6^+^ and Chrna2b^+^ MNs in the spinal cord. The size of the circles reflects the number of puncta per cell (# puncta) (*n* = 3 animals). **e**, RNAscope *in situ* hybridization of *pvalb6* and *neurod1* in slow–intermediate or fast MNs. **f**, Normalized soma position of Pvalb6^+^ and Neurod1^+^ MNs in the spinal cord. The size of the circles reflects the number of puncta per cell (# puncta) (*n* = 3 animals). **g**, RNAscope *in situ* hybridization of *neurod1* and *calb1* in slow–intermediate or fast MNs. **h**, Normalized soma position of Neurod1^+^ and Calb1^+^ MNs in the spinal cord (*n* = 4 animals). **i**, Number of puncta of each RNAscope probe in slow–intermediate and fast MNs (Two-tailed Mann–Whitney U test, ****p < 0.0001; Grin1b: *n* = 9 animals; Pvalb6: *n* = 10 animals; Chrna2b: *n* = 7 animals; Neurod1: *n* = 12 animals; Calb1: *n* = 9 animals). Source data

To determine whether the different clusters correspond to functional MN subtypes, we used RNAscope for specific genes that were highly enriched in each cluster combined with retrograde labeling of slow, intermediate or fast MNs^41^. These MNs can be identified based on the muscle type they innervate and their soma position in the spinal cord (Extended Data Fig. 1a). Two marker genes, *grin1b* and *pvalb6*, that were enriched in clusters MN_1_ and MN_2_ were coexpressed in MNs located in the ventrolateral aspect of the spinal cord (Fig. 2a–c,e,f). These MNs were identified as slow or intermediate, but not as fast MNs, based on the muscle they innervated and their soma size and position (Fig. 2d–f and Extended Data Fig. 1a). In contrast, two of the marker genes enriched in cluster MN_3_, *chrna2b* and *neurod1*, showed overlapping expression only in MNs located in the dorsomedial part of the spinal cord where fast MNs are located (Fig. 2g,h,j–l). These marker genes were not present in the ventromedial aspect, where slow and intermediate MNs are located (Fig. 2i,k,l). The fast MN identity of MN_3_ was further validated by the expression of *calb1*, which represents another highly expressed gene in this cluster (Fig. 2m,n and Extended Data Fig. 4g–i). Combinatorial expression analysis for these genes shows minimal overlap between MN_1_ and MN_2_ markers and those of MN_3_ markers, clearly defining the slow–intermediate MNs and the fast MNs, respectively (Extended Data Fig. 4a–i).

**Fig. 3:**
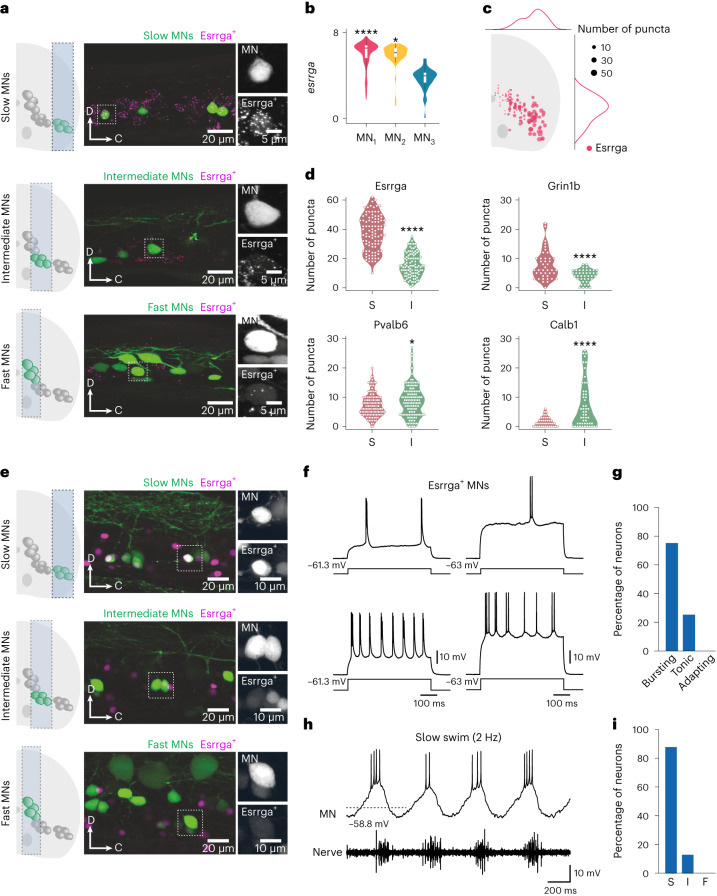
Characterization of Esrrga^+^ MNs. **a**, Lateral view of a spinal cord segment showing RNAscope in situ hybridization of *esrrga* in retrogradely labeled slow, intermediate and fast MNs. **b**, Violin plot of log-normalized expression of *esrrga* in the three MN clusters (MN_1_, *n* = 98; MN_2_, *n* = 51; MN_3_, *n* = 47; differential gene expression analysis, nonparametric Wilcoxon rank sum test with Bonferroni adjusted *P* value, *P = 0.014, ****P < 0.0001; boxes are bound by the 25th and 75th percentiles, the center line indicates the median and whiskers extend from minimum to maximum). **c**, Normalized distribution of Esrrga^+^ MNs in the spinal cord. **d**, Violin plots of the number of puncta of *esrrga* (*n* = 8 animals), *grin1b* (*n* = 9 animals), *pvalb6* (*n* = 9 animals) and *calb1* (*n* = 9 animals) in slow (S) and intermediate (I) MNs (two-tailed Mann–Whitney *U* test; *P = 0.0281, ***P = 0.0003, ****P < 0.0001). **e**, Lateral view of a spinal cord segment showing the expression of a fluorescence reporter driven by *esrrga* in a transgenic line in slow, intermediate and fast MNs. **f**, Two examples of Esrrga^+^ MNs firing in bursts of action potentials in response to depolarizing current injections. **g**, Percentage of Esrrga^+^ MNs with bursting, tonic and adapting firing patterns (*n* = 16 neurons). **h**, Recruitment of an Esrrga^+^ MN at slow swim frequencies. **i**, Percentage of Esrrga^+^ MNs recruited at slow (S), intermediate (I) or fast (F) swim frequencies (*n* = 16 neurons). Source data

Whereas MN_3_ could be identified as representing fast MN identity, clusters MN_1_ and MN_2_ exhibited overlapping gene expression. Nevertheless, these two clusters displayed differential expression levels of specific genes. In particular, the marker gene *pvalb6* showed higher expression in MN_2_ compared with MN_1_ (Fig. 2m). The MNs with the highest expression of *grin1b* were located primarily laterally, where slow MNs are typically found, whereas those with the highest expression of *pvalb6* were situated mainly medially, corresponding to intermediate MNs (Fig. 2n–p). Another gene, *esrrga*, also exhibited higher expression in MN_1_ than in MN_2_ (Fig. 3a,b). RNAscope in situ hybridization analysis showed a pronounced enrichment of *esrrga* in MNs located in the lateral part of the spinal cord (Fig. 3a,c). Additionally, the expression of *esrrga* and *grin1b* was significantly higher in slow MNs compared with intermediate MNs, whereas *pvalb6* and *calb1* expression was significantly higher in intermediate MNs (Fig. 3d). These results suggest that MN_1_ and MN_2_ share overlapping gene markers. However, these clusters could be associated with slow or intermediate MNs based on their lateromedial location in the spinal cord combined with their level of expression of specific genes.

To determine the functional identity of Esrrga^+^ MNs, we used a transgenic line in which the expression of a fluorescent reporter was driven by *esrrga*. The different MN subtypes were labeled retrogradely in this line and fluorescence was used as a proxy for the level of expression of *esrrga* (Fig. 3e). MNs with the highest expression of *esrrga* were located primarily in the ventrolateral aspect of the spinal cord, consistent with the typical location of slow MNs (Fig. 3e). Subsequently, we performed whole-cell patch-clamp recordings from Esrrga^+^ MNs to validate their functional identity. Most of the recorded Esrrga^+^ MNs fired in bursts of action potentials (Fig. 3f,g)—a characteristic feature of slow MNs in adult zebrafish^31,32^. Importantly, none of the recorded Esrrga^+^ MNs displayed an adapting firing pattern, which is known to be restricted to fast MNs in adult zebrafish (Fig. 3g). To further confirm the slow MN identity of Esrrga^+^ MNs, we determined their recruitment frequency during swimming and showed that almost all the recorded Esrrga^+^ MNs were recruited at slow swim frequencies (<3 Hz; Fig. 3h,i).

**Extended Data Fig. 5:**
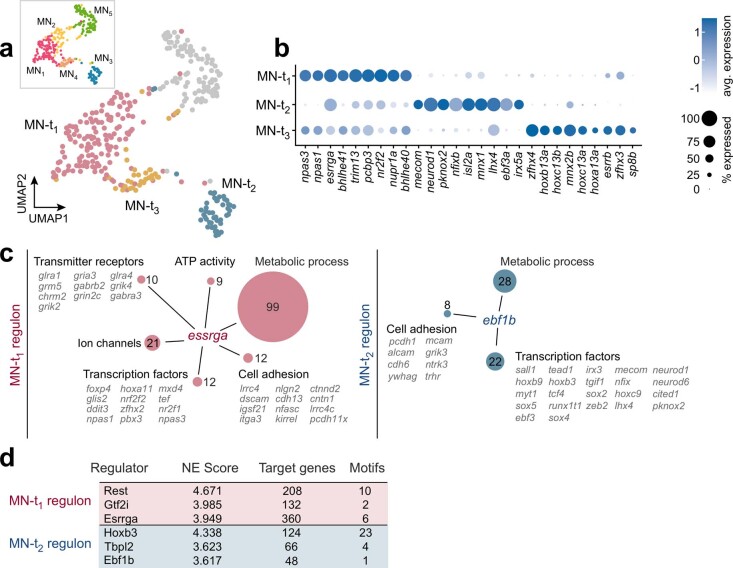
Analysis of TFs and regulons in the MN dataset. **a**, UMAP of three color-coded TF-defined MN clusters (MN-t_1-3_). The cluster in gray corresponds to the immature MN cluster (MN_4_). The inset shows whole dataset-defined clusters from Fig. 1. **b**, Examples of differentially expressed TFs in each TF-defined MN cluster. The size of the circle reflects the proportion of the cells expressing the gene in a cluster (% expressed), and the color intensity reflects its average expression level within that cluster (avg. expressed). **c**, Plots of highly enriched regulons in cluster MN-t_1_ (left) and MN-t_2_ (right). **d**, Table showing regulons with highest normal enrichment scores (NES) obtained by iRegulon analysis. A high NES ( > 3.0) indicates a potential regulator TF of a subset of target genes sharing specific RNA binding motifs.

To assess the importance of transcription factors (TFs) in defining the diversity of the MN subtypes, we clustered these MNs using exclusively the expression of TF genes. The resulting clusters (referred to as MN-t clusters) showed a striking correspondence with the clusters obtained from the whole transcriptome analysis (Extended Data Fig. 5). More specifically, the MN population comprised three TF-defined clusters, where MN_1_ and MN_2_ became merged into a single cluster MN-t_1_, while the clusters corresponding to fast (MN-t_2_) and caudal (MN-t_3_) MNs remained separated (Extended Data Fig. 5a). Each of these clusters was enriched with a selective set of TFs (Extended Data Fig. 5b). We next performed regulon analysis followed by GO to determine whether specific TFs play a regulatory role for slow–intermediate (MN-t_1_) and fast (MN-t_2_) MN clusters. This analysis revealed selective regulons comprising specific key TFs for each of the clusters (Extended Data Fig. 5c,d). One of the top-scoring regulons in the slow–intermediate cluster MN-t_1_ was *esrrga*, which interacted with a large network of predicted target genes (Extended Data Fig. 5d). Fast cluster MN-t_2_ was enriched in different regulons, and among the top ones was *ebf1b*, which forms a network with other predicted TF genes, including *neurod1* (Extended Data Fig. 5c,d).

These results show that the different functional MN subtypes can be captured molecularly based on their gene expression profile and validated electrophysiologically. While fast MNs are clearly separated from slow–intermediate MNs based on their TF and their overall gene expression, the assignment of slow versus intermediate identity is not recapitulated by TFs alone and becomes apparent based on the expression levels of specific genes.

### Molecular diversity of V2a INs

**Fig. 4:**
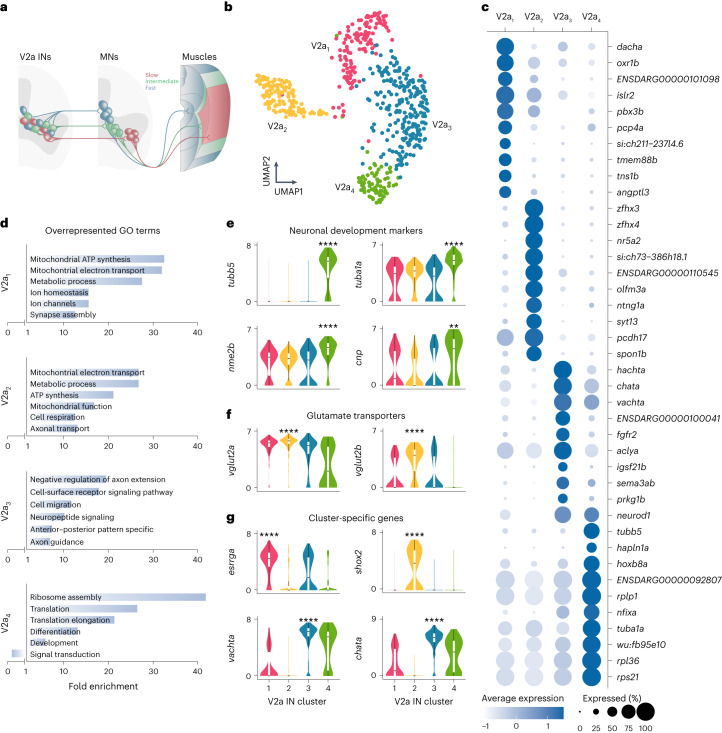
Molecular characterization of V2a IN diversity. **a**, Schematic showing the soma position and connectivity pattern of V2a INs and MNs in the spinal cord as well as the innervation and organization of axial muscles in adult zebrafish. **b**, V2a INs single-cell transcriptomes visualized with UMAP, color-coded for four molecularly defined clusters (V2a_1–4_, *n* = 593 cells; V2a_1_, *n* = 137; V2a_2_, *n* = 125; V2a_3_, *n* = 221; V2a_4_, *n* = 77). **c**, Examples of differentially expressed genes in each cluster. The size of the circle reflects the proportion (%) of the cells expressing the gene in a cluster, and the color intensity reflects its average expression level within that cluster. **d**, GO analysis of differentially expressed genes in each cluster. **e**, Log-normalized expression of neuronal development gene markers (differential gene expression analysis, nonparametric Wilcoxon rank sum test with Bonferroni adjusted *P* value, **P = 0.0027, ****P < 0.0001). **f**, Log-normalized expression of glutamate transporter genes (differential gene expression analysis, nonparametric Wilcoxon rank sum test with Bonferroni adjusted *P* value, ****P < 0.0001). **g**, Log-normalized expression of selected gene markers for each cluster (differential gene expression analysis, nonparametric Wilcoxon rank sum test with Bonferroni adjusted *P* value, ****P < 0.0001). In **e**–**g**, boxes are bound by the 25th and 75th percentiles, the center line indicates the median and whiskers extend from minimum to maximum.

The diversity of V2a INs was determined using scRNA-seq of Chx10^+^ neurons dissociated from 7-week-old zebrafish spinal cords of the *vsx2* transgenic line referred to as Tg(*chx10*:GFP)^42^. This line is known to capture all V2a IN subtypes of the three speed circuit modules (Fig. 4a)^18,19,24,43^. A total of 593 Chx10^+^ neurons passed quality control and were used for cluster analysis. This analysis revealed four molecularly distinct clusters, of which V2a_4_ showed distinct characteristics of immature neurons (Fig. 4b,c). This cluster was highly enriched with genes related to development, differentiation and RNA translation, but was less enriched with genes for glutamate synaptic transmission (Fig. 4d–f), supporting the immature nature of neurons of this cluster; hence, it was not analyzed further.

GO analysis showed that clusters V2a_1_ and V2a_2_, but not cluster V2a_3_, had overexpression of genes related to high metabolic activity associated with sustained firing properties (Fig. 4d). While all the three clusters expressed marker genes for glutamate synaptic transmission (Fig. 4f), they were selectively enriched with specific marker genes (Fig. 4g). Among the differentially expressed genes, V2a_1_ was enriched with *esrrga*, V2a_2_ with *shox2* and V2a_3_ with cholinergic transmission marker genes such as *chata* and *vachta* (Fig. 4g).

**Extended Data Fig. 6:**
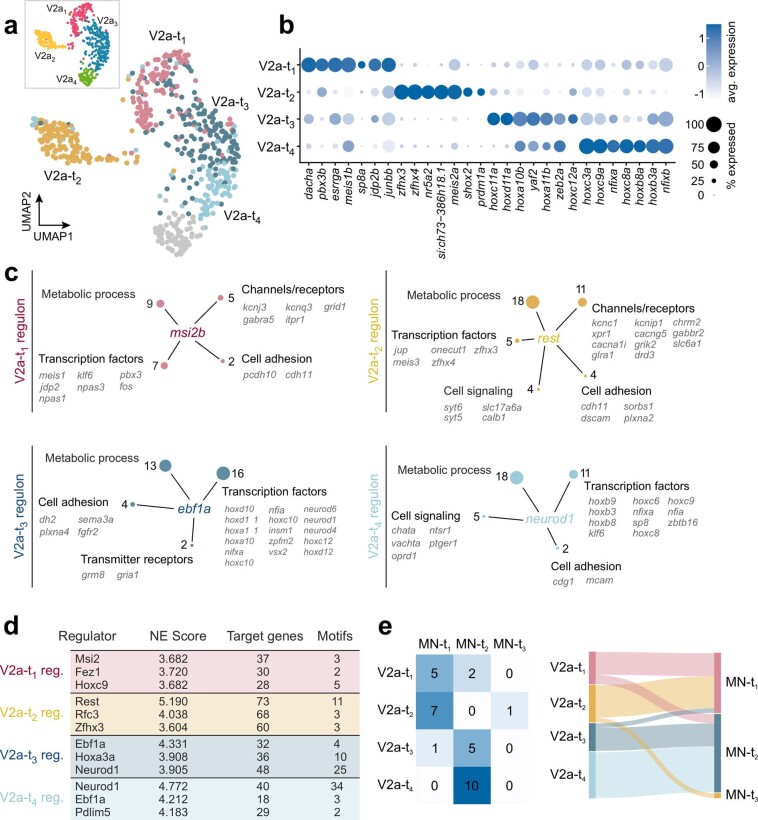
Analysis of TFs and regulons in the V2a IN dataset. **a**, UMAP of four color-coded TF-defined V2a IN clusters (V2a-t_1-4_). The cluster in gray corresponds to the immature V2a IN cluster (V2a_4_). The inset shows the whole dataset-defined clusters from Fig. 1. **b**, Examples of differentially expressed TFs in each TF-defined V2a IN cluster. The size of the circle reflects the proportion of the cells expressing the gene in a cluster (% expressed), and the color intensity reflects its average expression level within that cluster (avg. expressed). **c**, Plots of highly enriched regulons in each TF-defined V2a IN cluster. **d**, Table showing regulons with highest normal enrichment scores (NES) obtained by iRegulon analysis. A high NES (>3.0) indicates a TF that most likely acts as a regulator of a subset of target genes sharing specific RNA binding motifs. **e**, Heatmap (left) and Sankey plot (right) of differentially expressed genes shared by different TF-defined V2a IN and MN clusters.

The importance of TFs in determining the diversity of the V2a IN subtypes was assessed by reclustering these neurons exclusively by the expression of TF genes. This analysis resulted in four clusters (V2a-t_1–4_) (Extended Data Fig. 6a). V2a-t_1_ and V2a-t_2_ showed striking correspondence with the original clusters V2a_1_ and V2a_2_, respectively. However, the original V2a_3_ split into two closely related clusters, V2a-t_3_ and V2a-t_4_. Each cluster was defined by overexpression of several specific TFs (Extended Data Fig. 6b). Furthermore, regulon analysis identified cluster specific regulons with key TFs interacting with different target genes related to neuronal function, connectivity and transcription (Extended Data Fig. 6c,d). The slow and intermediate clusters V2a-t_1_ and V2a-t_2_ had different top-scoring regulons that interacted with different TFs, channel and receptor genes (Extended Data Fig. 6c,d). The top regulons of the fast clusters V2a-t_3_ and V2a-t_4_, *ebf1a* and *neurod1*, form a network with other predicted TF genes, including *vachta* (Extended Data Fig. 6c,d). Interestingly, the two top regulons of fast V2a INs are shared with the fast MN cluster MN-t_2_.

Overall, these results show that the diversity of V2a INs can be revealed by the differential expression of TFs alone with three main clusters defined by selective marker genes (*esrrga*, *shox2* or *vachta*).

### V2a IN clusters represent functional subtypes

**Fig. 5:**
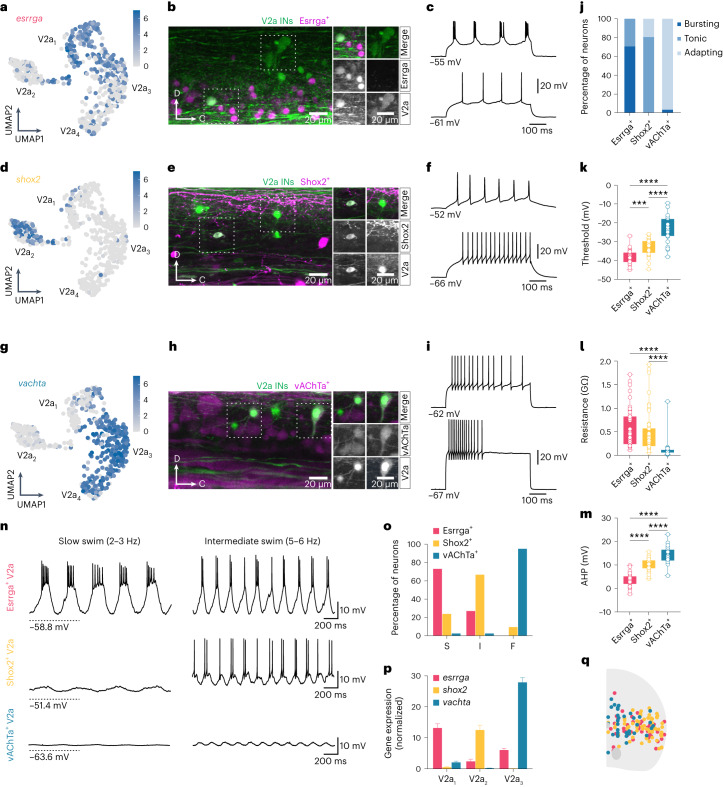
Electrophysiological and functional validation of V2a IN molecular clusters. **a**, UMAP of log-normalized expression of *esrrga* in V2a IN clusters. **b**, Expression of Essrga and Chx10 in a spinal segment. **c**, Examples of bursting (top) or tonic (bottom) firing Esrrga^+^ V2a INs. **d**, UMAP log-normalized expression of *shox2* in V2a IN clusters. **e**, Expression of Shox2 and Chx10 in a spinal segment. **f**, Examples of tonic firing Shox2^+^ V2a INs. **g**, UMAP log-normalized expression of *vachta* in V2a IN clusters. **h**, Expression of vAChTa and Chx10 in a spinal segment. **i**, Examples of adapting firing vAChTa^+^ V2a INs. **j**, Percentage of Esrrga^+^, Shox2^+^ or vAChTa^+^ V2a INs with bursting, tonic or adapting firing (*n* = 43 Esrrga^+^, *n* = 53 Shox2^+^; *n* = 28 vAChTa^+^ V2a INs). **k**, Firing threshold of the three V2a INs subtypes (one-way ANOVA with Tukey’s post hoc multiple comparisons, ***P = 0.0009 ****P < 0.0001; *n* = 23 vAChTa^+^; 28 Shox2^+^; 32 Esrrga^+^ V2a INs). **l**, Input resistance of the three V2a INs subtypes (Kruskal–Wallis test with Dunn’s post hoc multiple comparisons, ****P < 0.0001; *n* = 44 vAChTa^+^; *n* = 47 Shox2^+^; *n* = 47 Esrrga^+^ V2a INs). **m**, AHP amplitude of the three V2a INs subtypes (one-way ANOVA with Tukey’s post hoc multiple comparisons, ****P < 0.0001; *n* = 23 vAChTa^+^; *n* = 34 Shox2^+^; *n* = 30 Esrrga^+^ V2a INs). **n**, Top, an Esrrga^+^ V2a IN recruited at slow and intermediate swim frequencies. Middle, a Shox2^+^ V2a IN recruited at intermediate swim frequencies. Bottom, a vAChTa^+^ V2a IN not recruited at slow and intermediate swim frequencies. **o**, Percentage of Esrrga^+^, Shox2^+^ or vAChTa^+^ V2a INs identified as slow (S), intermediate (I) or fast (F) (*n* = 31 Esrrga^+^, *n* = 40 Shox2^+^; *n* = 38 vAChTa^+^ V2a INs). **p**, Averaged expression *esrrga*, *shox2* and *vachta* in V2a IN clusters (mean ± s.e.m.; V2a_1_: *n* = 137; V2a_2_: *n* = 125; V2a_3_: *n* = 221). **q**, Soma position of Esrrga^+^, Shox2^+^ and vAChTa^+^ V2a INs. In **k**–**m**, boxes are bound by the 25th and 75th percentiles and whiskers extend from minimum to maximum. Source data

**Extended Data Fig. 7:**
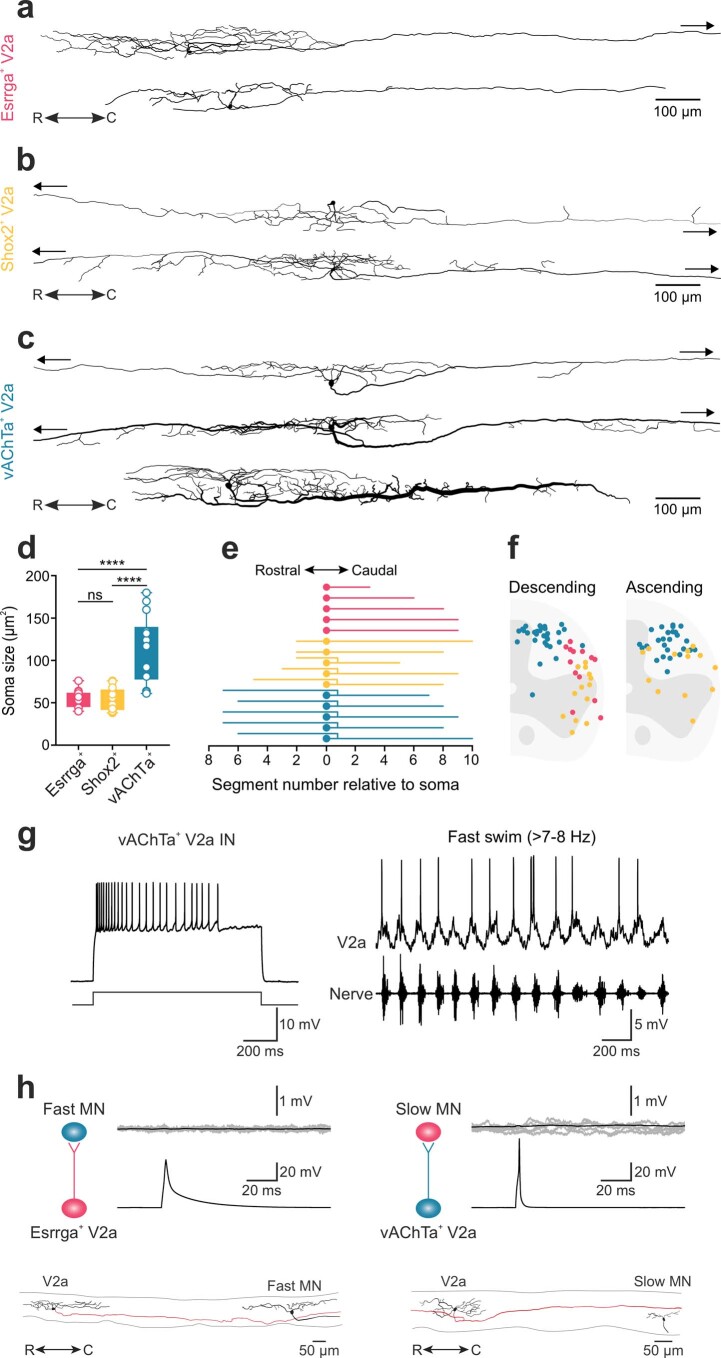
Morphological features of V2a IN subtypes. **a**, Esrrga^+^ V2a INs displayed only descending axonal projections. Arrow indicates continuing axonal projection. **b**, Lateral view of the morphology of two examples of Shox2^+^ V2a INs. **c**, vAChTa^+^ V2a INs mostly displayed bidirectional axonal projections. A specialized vAChTa^+^ V2a IN subtype showed located axonal projection and corresponds to the previously described escape V2a IN^41^. **d**, Box and whiskers plot of soma sizes of the three V2a INs subtypes (one-way ANOVA with Tukey’s post hoc multiple comparisons, ****p < 0.0001) (*n* = 12 Esrrga^+^; *n* = 12 Shox2^+^; *n* = 14 vAChTa^+^ V2a INs). **e**, Length of the axonal projections of Esrrga^+^ (magenta), Shox2^+^ (yellow) and vAChTa^+^ (blue) V2a INs (*n* = 5 neurons per subtype). **f**, V2a IN axons projected along topographically organized paths over the dorso-ventral and medio-lateral aspects of the spinal cord (*n* = 13 Esrrga^+^: magenta; *n* = 13 shox2^+^: yellow; *n* = 30 vAChTa^+^: blue). **g**, Recordings of a fast V2a IN displaying adapting firing that was recruited only at fast swimming frequency (>7-8 Hz). **h**, Top left: Example of dual patch-clamp recordings showing the lack of monosynaptic connection between a slow, Esrrga^+^ V2a IN and a fast MN. Bottom left: Morphological reconstruction of the recorded V2a IN-MN pair (black: soma and dendrites; red: V2a IN axons). Top right: Example of dual patch-clamp recording showing the absence of connections between a fast, vAChTa^+^ V2a IN and a slow MN. Bottom right: Morphological reconstruction of the recorded V2a IN-MN pair (black: soma and dendrites; red: V2a IN axons). Source data

To examine whether the molecularly defined V2a IN clusters represent different functional subtypes within the spinal locomotor circuits, we performed electrophysiological analysis. We used transgenic lines in which a fluorescent reporter (GFP or red fluorescent protein (RFP)) expression is driven by *esrrga*, *shox2* or *vachta* that were crossed either with Tg(*chx10*:RFP) or Tg(*chx10*:GFP). The Esrrga^+^ V2a INs were restricted mostly to cluster V2a_1_ (Fig. 5a,b) and most of them displayed pacemaker properties and fired in bursts of action potentials in response to depolarizing current injections (Fig. 5c,j). These Esrrga^+^ V2a INs were of small size and displayed only unidirectional descending axonal projections in the lateral aspect of the spinal cord (Extended Data Fig. 7a,d–f). The Shox2^+^ V2a INs were confined to cluster V2a_2_ (Fig. 5d,e) and fired mostly tonically when they were depolarized with current injections (Fig. 5f,j). These V2a INs also had small soma size but displayed bidirectional axonal projections with a longer descending and shorter ascending projection also running in the lateral aspect of the spinal cord (Extended Data Fig. 7b,d–f). The vAChTa^+^ V2a INs were restricted to cluster V2a_3_ (Fig. 5g,h), were characterized by a strong adaptation and fired mostly at the beginning of the current injection (Fig. 5i,j). These V2a INs had a large soma size and always displayed long bidirectional axonal projections with a main descending axon running medially in the spinal cord that gave rise to an ascending collateral projection (Extended Data Fig. 7c–f). In addition, previously described local cholinergic V2a INs embedded in the escape circuit (esV2a)^44^ were also captured in the vAChTa^+^ V2a IN population (Extended Data Fig. 7c).

In addition to differences in their firing properties and morphology, these three V2a INs subtypes defined by molecular clustering also displayed clear differences in their excitability. The most excitable were the Esrrga^+^ V2a INs (cluster V2a_1_), which had the lowest firing threshold, highest input resistance and smallest afterhyperpolarization (AHP; Fig. 5k–m). The Shox2^+^ V2a INs (cluster V2a_2_) were also characterized by high excitability, albeit to a lesser extent than the Esrrga^+^ neurons (Fig. 5k–m). In contrast, the vAChTa^+^ V2a INs (cluster V2a_3_) were the least excitable and had the highest firing threshold, lowest input resistance and largest AHP (Fig. 5k–m).

The difference in excitability of the three V2a IN subtypes mirrored the sequential order of their recruitment with increasing swimming speeds. Most of the Esrrga^+^ V2a INs were recruited at the slowest swim frequencies (<4 Hz) and maintained their recruitment as the speed increased (Fig. 5n). The Shox2^+^ V2a INs were recruited mostly at intermediate swim speeds (4–7 Hz) (Fig. 5n). vAChTa^+^ correspond to fast V2a INs as they were never recruited at slow or intermediate frequencies up to 7 Hz (Fig. 5n), and they become recruited at higher frequencies (>7–8 Hz) (Extended Data Fig. 7g)^18^. The preferential speed-dependent recruitment of the three V2a INs subtypes (slow, intermediate and fast) (Fig. 5o) matched the expression levels of their selective marker genes of the three transcriptionally defined V2a IN clusters (V2a_1–3_) (Fig. 5p). RNAscope analysis of *esrrga*, *shox2* and *vachta* in Tg(*chx10*:GFP) revealed that the soma of V2a INs of the three clusters did not show any preferential position as they were intermingled in the spinal cord (Fig. 5q). These results show a tight correspondence between the anatomical, electrophysiological and functional properties of V2a IN subtypes^18,19,23,24^ and their distinct molecular profiles. Slow V2a INs comprise cluster V2a_1_, intermediate comprise cluster V2a_2_ and fast V2a INs comprise cluster V2a_3_.

### Early differentiation of MNs and V2a INs

**Extended Data Fig. 8:**
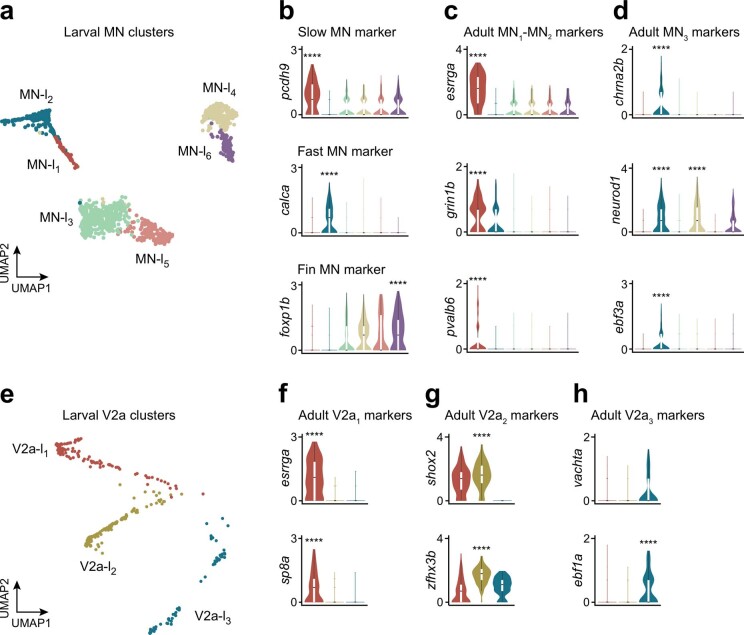
Molecular diversity of larval MNs and V2a INs. **a**, UMAP of six color-coded, molecularly defined larval MN clusters (MN-l_1-6_, *n* = 1354 cells; MN-l_1_: *n* = 91; MN-l_2_: *n* = 212; MN-l_3_: *n* = 467; MN-l_4_: *n* = 251; MN-l_5_: *n* = 208; MN-l_6_: *n* = 125). **b**, Log-normalized expression levels of known gene markers of slow, fast and fin MN in zebrafish (differential gene expression analysis, non-parametric Wilcoxon rank sum test with Bonferroni adjusted p-value, ****p < 0.0001). **c**, Log-normalized expression levels in larval MN clusters of gene markers of slow–intermediate MN in adult zebrafish (MN_1_ and MN_2_) (differential gene expression analysis, non-parametric Wilcoxon rank sum test with Bonferroni adjusted p-value, ****p < 0.0001). **d**, Log-normalized expression levels in larval MN clusters of gene markers of fast MN in adult zebrafish (MN_3_) (differential gene expression analysis, non-parametric Wilcoxon rank sum test with Bonferroni adjusted p-value, ****p < 0.0001). **e**, UMAP of three color-coded, molecularly defined larval V2a IN clusters (V2a-l_1-3_, *n* = 316 cells; V2a-l_1_: *n* = 108; V2a-l_2_: *n* = 128; V2a-l_3_: *n* = 80). **f**, Log-normalized expression levels in larval V2a IN clusters of gene markers for slow V2a INs in adult zebrafish (V2a_1_) (differential gene expression analysis, non-parametric Wilcoxon rank sum test with Bonferroni adjusted p-value, ****p < 0.0001). **g**, Log-normalized expression levels in larval V2a IN clusters of gene markers for intermediate V2a INs in adult zebrafish (V2a_2_) (Differential gene expression analysis, non-parametric Wilcoxon rank sum test with Bonferroni adjusted p-value, ****p < 0.0001). **h**, Log-normalized expression levels in larval V2a IN clusters of gene markers for fast V2a INs in adult zebrafish (V2a_3_) (differential gene expression analysis, non-parametric Wilcoxon rank sum test with Bonferroni adjusted p-value, ****p < 0.0001).

To determine whether the transcriptional differentiation of MNs and V2a INs is established early during development, we analyzed available scRNA-seq data from 4 days postfertilization larvae^45^. Larval MN segregated into six clusters (MN-l_1–6_) (Extended Data Fig. 8a). Two of these clusters expressed known markers of either slow (MN-l_1_) or fast (MN-l_2_) MNs, whereas the other four clusters (MN-l3-6) expressed a marker of fin MNs, *foxp1b* (ref. ^46^) (Extended Data Fig. 8b). Larval MN-l_1_ was highly enriched in gene markers of adult MN clusters (MN_1_ and MN_2_; *esrrga*, *grin1b*, *pvalb6*) corresponding to slow–intermediate MNs (Extended Data Fig. 8c), whereas larval MN-l_2_ was enriched in gene markers specific to the adult fast MN cluster (MN_3_; *chrna2b*, *neurod1*, *ebf3a*) (Extended Data Fig. 8d). Similarly, this analysis revealed three larval V2a IN clusters (V2a-l_1–3_) (Extended Data Fig. 8e). Each of these clusters was selectively enriched in adult gene markers of either slow (V2a_1_; *esrrga*, *sp8a*), intermediate (V2a_2_; *shox2*, *zfhx3b*) or fast (V2a_3_; *vachta*, *ebf1a*) V2a INs (Extended Data Fig. 8f–h). These results suggest that, early during development, MNs are differentiated into two main molecularly defined subtypes, whereas V2a INs are already segregated into three molecularly distinct subtypes.

### Molecular underpinnings of circuit modules

**Fig. 6:**
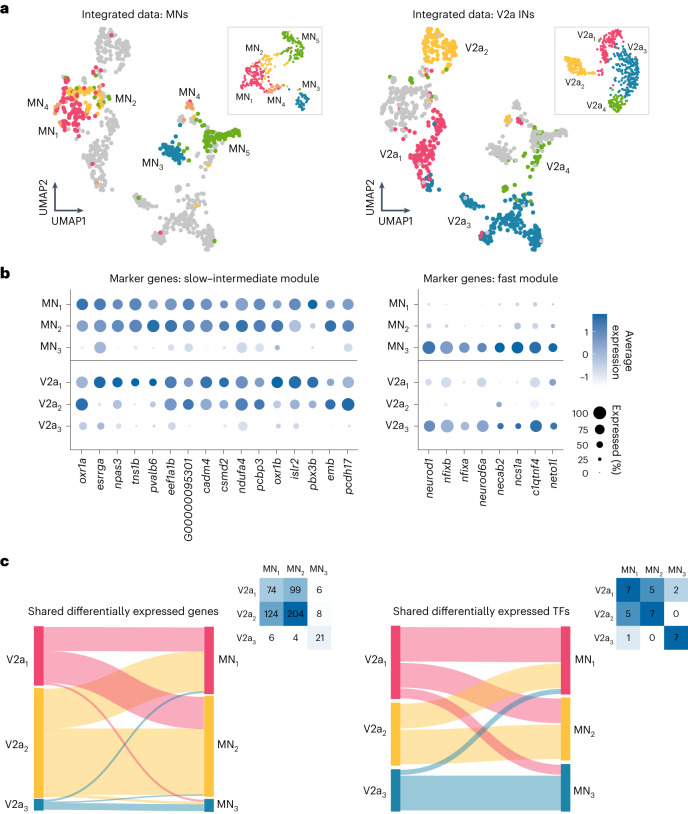
Shared features of functional speed modules across spinal populations. **a**, Left, UMAP of the MN and V2a IN integrated dataset, color-coded for original MN clusters (inset). Right, UMAP of the integrated dataset, color-coded for original V2a IN clusters (inset). **b**, List of differentially expressed genes in the different circuit modules. Left, slow and intermediate modules; right, fast module. The size of the circle reflects the proportion (%) of the cells expressing the gene in a cluster, and the color intensity reflects its average expression level within that cluster. **c**, Top, Sankey plot of overall differentially expressed genes (left) and differentially expressed TFs (right) shared by different modules of V2a IN and MN clusters. Insets, data are presented as a heatmap with number of genes.

V2a INs and MNs are known to be connected in a specific organization to form three circuit modules that drive locomotion at slow, intermediate and fast speeds^18,24^. However, it is not known whether each of these circuit modules has a common molecular signature. To address this question, we first analyzed the integrated MN and V2a IN transcriptome, which allows the identification of shared molecular features among the two neuronal populations^47^. The integrated dataset revealed a distinct pattern with the MN and V2a IN clusters belonging to the same speed module being located in close proximity in the transcriptomic space (Fig. 6a). All the clusters comprising the slow and intermediate speed modules across both populations occupied the left half of the transcriptome map (Fig. 6a). The fast MN cluster together with fast V2a INs, and the immature neurons of both neuronal populations occupied the right half of the map (Fig. 6a). Furthermore, there were several genetic markers for the slow/intermediate or fast module that were enriched both in MN and V2a IN datasets (Fig. 6b). These include TFs (*esrrga*, *neurod1*, *nfixa, nfixb*); genes that regulate neuronal physiology (*oxr1a, oxr1b;* oxidation resistance genes for the metabolically active slow module); and cell adhesion molecules that could be important in the establishment and maintenance of the proper neuronal connectivity (*csmd2*, *pcdh17*, *ncs1a*, *c1qtnf4*).

To determine whether the differentially expressed genes in each of the V2a IN and MN clusters capture their modular circuit identity, we compared the gene expression profiles of each functional cluster of the two separate datasets. Analysis of the overall differentially expressed gene set and TFs showed a clear correspondence between V2a IN and MN clusters belonging to the same speed module, with some overlap between slow and intermediate clusters (Fig. 6c). A similar analysis was performed on V2a INs and MNs that were clustered exclusively by their TF expression (Extended Data Figs. 5a,b and 6a,b). This analysis also showed a clear correspondence between TF-defined V2a IN and MN clusters according to their respective speed module (Extended Data Fig. 6e). These results show that the three V2a IN-MN speed modules, previously defined electrophysiologically^18,19,23,24^, are each characterized by common molecular signatures.

**Fig. 7:**
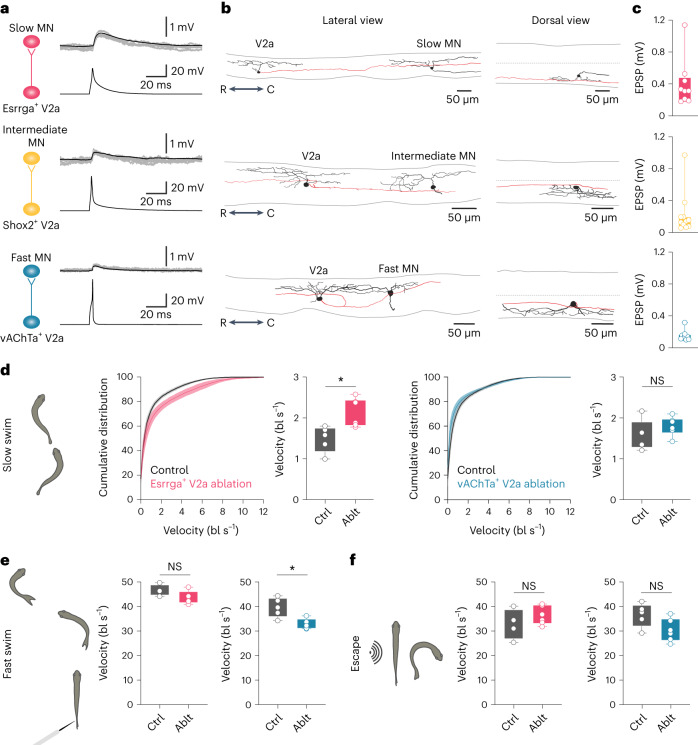
Functional connectivity between transcriptomically defined V2a IN and MN subtypes. **a**, Monosynaptic connections between presynaptic Esrrga^+^ (top), Shox2^+^ (middle) or vAChTa^+^ (bottom) V2a INs and postsynaptic slow/Esrrga^+^, intermediate or fast MNs, respectively. **b**, Morphological reconstruction of the connected V2a IN–MN pairs (black, soma and dendrites; red, V2a IN axons). **c**, EPSP amplitude of V2a IN–MN connected pairs of each module (*n* = 9, top; *n* = 11, middle; *n* = 9, bottom). **d**, Two-photon ablation of slow/Esrrga^+^ but not of fast/vAChTa^+^ V2a INs affected slow explorative swimming. Graphs show the cumulative distribution or average swimming velocity in control (Ctrl) and zebrafish with ablated (Ablt) slow/Esrrga^+^ (magenta, left) or fast/vAChTa^+^ (blue, right) V2a INs (mean ± s.e.m.; two-tailed Student’s *t*-test, *P = 0.0111; Esrrga^+^ V2a IN ablation, *n* = 5 controls and *n* = 6 ablated; vAChTa^+^ V2a IN ablation, *n* = 6 controls and *n* = 6 ablated; bl, body length). **e**, Two-photon ablation of fast/vAChTa^+^ (blue, right), but not slow/Esrrga^+^ (magenta, left) V2a INs affected touch-induced fast swimming (mean ± s.e.m.; two-tailed Student’s *t*-test, *P = 0.0102; Esrrga^+^ V2a IN ablation, *n* = 5 controls and *n* = 6 ablated; vAChTa^+^ V2a IN ablation, *n* = 6 controls and *n* = 6 ablated). **f**, Sound-induced escape behavior was not affected by ablation of slow/Esrrga^+^ (magenta, left) or fast/vAChTa^+^ (blue, right) V2a INs (mean ± s.e.m.; two-tailed Student’s *t*-test, *P* > 0.05; Esrrga^+^ V2a IN ablation, *n* = 5 controls and *n* = 6 ablated; vAChTa^+^ V2a IN ablation, *n* = 6 controls and *n* = 6 ablated). In **c**–**f**, boxes are bound by the 25th and 75th percentiles, whiskers extend from minimum to maximum. Source data

Next, we performed dual patch-clamp recordings from V2a INs of each transcriptionally defined cluster and slow, intermediate or fast MNs to examine their functional connectivity. To target V2a INs of each cluster, we used transgenic lines in which the expression of a fluorescent reporter was driven by *esrrga*, *shox2* or *vachta*, and crossed with *chx10* transgenic lines (see above). Retrograde labeling of fluorescent dyes into the respective muscles allowed us to target slow, intermediate and fast MNs. Esrrga^+^ V2a INs (cluster V2a_1_) exhibited reliable monosynaptic connections with slow, Esrrga^+^ MNs (Fig. 7a,c; top). Reconstruction of the connected pairs revealed that the axons of Esrrga^+^ V2a IN specifically targeted the dendrites of slow MN (Fig. 7b; top). Similarly, Shox2^+^ V2a INs (cluster V2a_2_) displayed consistent monosynaptic connections with intermediate MNs through axo-dendritic contacts (Fig. 7a–c; middle). vAChTa^+^ V2a INs (cluster V2a_3_) were found to be connected to fast MNs (Fig. 7a, c; bottom). In contrast to slow and intermediate V2a INs, the axon collaterals of fast V2a INs targeted the soma of fast MNs (Fig. 7b; bottom), as described previously^23^. Furthermore, there were no connections between slow Esrrga^+^ V2a INs and fast MNs, nor between fast vAChTa^+^ V2a INs and slow MNs (Extended Data Fig. 7h). These results show selective and reliable connections of transcriptionally defined V2a INs with MNs of the corresponding module.

Finally, to assess the functional impact of transcriptionally defined V2a INs on swimming behavior, we performed loss-of-function experiments combined with behavioral analysis. Ablation of slow, Esrrga^+^ V2a INs selectively impaired slow explorative swimming, resulting in a shift towards higher speeds (Fig. 7d). Conversely, ablation of fast, vAChTa^+^ V2a INs affected touch-induced fast swimming by decreasing the maximum speed (Fig. 7e). Ablation of slow, Esrrga^+^ or fast, vAChTa^+^ V2a INs did not affect sound-induced escape behavior (Fig. 7f).

## Discussion

The diversity and flexibility of motor actions is a result of the assembly, connectivity and properties of the underlying circuits in the central nervous system. For locomotion, these circuits are located in the spinal cord and the constituent neurons emerge during development through specific molecular programs to form different cardinal neuronal classes. These principal classes are heterogenous, and contain a diversity of neuron types, but it has been difficult to capture the importance of this diversity at the functional level. In this study, we used scRNA-seq in adult zebrafish to link the molecular diversity of MNs and V2a INs with the modular circuit organization that is responsible for changes in locomotor speed. We show that each neuronal population comprises three specific subtypes defined by key molecular features and that correspond to neurons underlying locomotion at slow, intermediate and fast speeds. Furthermore, our analysis reveals molecular signatures that define each of the three V2a IN–MN circuit speed modules. This study uncovers the molecular underpinnings of neuronal diversity and how they relate to the function of locomotor circuits in adult zebrafish.

Several key findings emerge from this study. First, three molecularly defined adult MN subtypes emerge that correspond to those innervating slow, intermediate or fast muscles^31,32^. Second, there was a substantial overlap in transcriptional profiles between slow and intermediate, while fast MNs displayed distinct molecular features. The expression of TF genes alone did not separate slow and intermediate MNs into distinct clusters. These clusters were instead distinguished based on their expression levels of specific genes. Interestingly, transcriptomic analysis during larval stages revealed two clusters of axial MNs that were enriched with marker genes for either slow–intermediate or fast adult MNs. This suggests that the segregation between slow and fast MNs occurs early during development. However, the separation of slow and intermediate MNs in the adult may involve experience-dependent plasticity and/or other developmental changes that are related to their circuit connectivity and the maturation of the muscle types they innervate.

Similarly, adult V2a INs were also divided into three molecularly distinct subtypes whose functional and morphological features matched those recruited at slow, intermediate or fast swimming speeds. These three V2a IN subtypes were already differentiated at early developmental stages. The diversity of MNs and V2a INs could be captured by their expression of specific TFs, which could have key regulatory roles. Finally, our analysis revealed a molecular logic associating the diversity of V2a INs and MNs to their connectivity into three speed circuits. Our results indicate that key molecular features are shared across classes, and they correlate with their common functional and circuit identity. Thus, conserved elements of connection specificity underlying locomotor circuit modularity correlate with common sets of TFs within V2a INs and MNs. These results suggest that the molecular signature for the modular circuit organization revealed here could represent a principle by which circuits are defined as functional entities, including those driving locomotion in mammals.

In mice, recent studies have used transcriptional profiling to show heterogeneity among ventral spinal neuronal classes^15–17,27–30,48–52^. Considerable diversity was demonstrated within the V2a IN population, with differentially expressed molecular markers reflecting the spatial location, axonal projections and rostrocaudal position along the spinal cord^17^. Moreover, in some studies in mice, V2a INs were segregated into two to four clusters^17,48,50–52^. Some of the markers (for example, *shox2*, *zfhx3b*, *neurod2*, *nfib* and *esrrga*) of the V2a IN clusters revealed in mice are similar to those that we now reveal in V2a INs of adult zebrafish. This further supports the notion that there are conserved molecular markers defining the diversity within the V2a IN population.

Analysis of the MN transcriptome in mice has shown that the molecular diversity of MNs relates to different MN pools innervating distinct muscles, rather than distinguishing specific MN subtypes innervating slow versus fast muscle fibers, which are mixed in many of these muscles^26,27,29^. In zebrafish, the situation is more advantageous with a separate location of slow, intermediate and fast muscle fibers and their respective MNs in the medial motor column. Although, in mice, MN subtypes were not segregated into different clusters, some known marker genes related to slow versus fast were enriched in different neurons within the same cluster^27^. Recent studies in embryonic and larval zebrafish used single-cell RNA profiling to identify molecular signatures for MN subtypes and their specification at early developmental stages^28,45^. Our study in adult zebrafish reveals that the three subtypes of functionally defined MNs (slow, intermediate and fast) are delineated by distinct molecular features and identified several additional molecular markers for slow, intermediate and fast MNs that could be used to guide studies in mammals.

Our transcriptomic analysis reveals general circuit principles based on spinal neuron diversity beyond the basic cardinal populations. The neuronal populations that form the locomotor network have been shown to display close or overlapping distributions in the transcriptomic space with overlapping gene expression patterns^51^. In addition, conserved genetic signatures define the axonal projection pattern among different neuronal classes^50^ raising the possibility for orthogonal marker modules across neuronal spinal classes. Our study not only links the molecular identity of neuronal types to their function but also uncovers orthogonal transcriptomic rules related to their modular circuit organization controlling locomotion. Thus, characterizing how the molecular diversity of MN and V2a INs relates to their function, connectivity and behavior provides groundtruth insights not only into the circuit mechanisms of locomotor flexibility but also for charting circuits for motor actions in general.

## Methods

### Animals

Zebrafish (*Danio rerio*) were raised and housed according to established protocols in the Comparative Medicine Biomedicum facility, Karolinska Institutet. In this study, adult animals 7–11 weeks old of either sex were used. All transgenic lines used in this study were obtained from the Higashijima laboratory. The *islet-1* transgenic line Tg(*isl1a*:GFP) (ZFIN: ZDB-TGCONSTRCT-070117-161), in which GFP expression driven by the promoter of *isl1a* is found in MNs and other INs, was used to sequence the transcriptome of MNs. The *vsx2* transgenic line referred to as Tg(*chx10*:GFP) (ZFIN: ZDB-GENO-010924-10), in which GFP is expressed in V2a INs, was used to sequence the transcriptome of the V2a INs and to perform RNAscope experiments. The AB wild type strain (ZFIN: ZDB-GENO-960809-7) was used to perform RNAscope experiments in which MNs were labeled by dye injections in the muscles. The selective targeting of Esrrga^+^ V2a INs was obtained by crossing Tg(*esrrga*-hs:loxP-RFP-loxP-GFP) and Tg(*chx10*:GFP). The selective targeting of Shox2^+^ V2a INs was obtained by crossing Tg(*shox2*:Gal4), Tg(UAS:GFP) and Tg(*chx10*-loxP-dsRed-loxP-GFP) (referred to as Tg(*chx10*:RFP), ZFIN: ZDB-TGCONSTRCT-070117-143). The selective targeting of vAChTa^+^ V2a INs was obtained by crossing Tg(*vachta*:GFP) and Tg(*chx10*:RFP). All experimental procedures followed European Union guidelines and were approved by the Animal Research Ethical Committee in Stockholm (Stockholms djurförsöksetiska nämnd, Dnr 6517-2019 and Dnr 19429-2022).

### Tissue dissociation, FACS and scRNA-seq

Adult animals (7 weeks old) of either sex were deeply anesthetized in a slush of frozen extracellular solution containing: 134 mM NaCl, 2.9 mM KCl, 2.1 mM CaCl_2_, 1.2 mM MgCl_2_, 10 mM HEPES and 10 mM glucose, with pH 7.8 adjusted with NaOH and osmolarity of 290 mOsm. The spinal cord was dissected quickly in the slush of frozen extracellular solution and collected. Two samples were prepared from the Tg(*isl1a*:GFP) line, and two samples were prepared from the Tg(*chx10*:GFP) line. For each sample, six to ten intact isolated spinal cords were incubated in 1 ml of DMEM/F-12 medium (Thermo Fisher, catalog no. 11039021, osmolarity adjusted to 280–290 mOsm) containing papain 10 U ml^−^^1^ (Worthington Biochemical, catalog no. LK003178) on a heated shaker at 37 °C for 15 min. DMEM/F-12 (1 ml, 280–290 mOsm) was added to stop the enzymatic reaction. The sample was centrifuged at 300*g* at 4 °C for 5 min, and then resuspended in 0.5 ml of DMEM/F-12 (280–290 mOsm) after removal of the supernatant. Following mechanical trituration using fire-polished Pasteur pipettes, the cell suspension was filtered through a cell strainer (40 μm). The sample was kept at room temperature for 20 min after the addition of 0.1 ml of the nuclear DNA stain DRAQ5 (Thermo Fisher, catalog no. 65-0880-92). Using FACS, cells positive for GFP and DRAQ5 in each sample were sorted into a 384-well plate containing a mild hypotonic lysis buffer (0.2% Triton X-100, 2 U ml^−^^1^ RNase inhibitor) and immediately snap-frozen on ice, then stored at −80 °C. RNA in single isolated cells was sequenced by the Eukaryotic Single Cell Genomics Facility at SciLifeLab, Stockholm, following the previously published protocol Smart-seq2 (refs. ^35,36^) using 150,000 reads per cell.

### Transcriptome analysis

The reads from each sequenced cell were mapped to the zebrafish reference genome ‘Danio_rerio, Ensembl, GRCz11’ using STAR (v.2.5.3a)^53^. The resulting bam files were filtered to keep only uniquely mapped reads. Most of the subsequent analysis was performed in R (v.4.0.5, R Core Team, 2022) using the Seurat package (v.4.0.2)^47^. Analysis of each dataset was conducted separately. The two samples of the Tg(*isl1a*:GFP) line dataset were integrated using the IntegrateData (30 dimensions, all genes used) Seurat function to avoid sample bias. In both datasets, we excluded genes that were expressed in fewer than three cells, and cells that had more than 30% reads from mitochondrial genes or from spike-ins, or a number of genes higher than 9,000 or lower than 2,000. After this quality control, the Tg(*isl1a*:GFP) line dataset contained 606 cells and the Tg(*chx10*:GFP) line dataset contained 593 cells. The gene expression measurements within each cell were scaled by a constant factor then log-normalized (gene counts for each cell are divided by the total counts for that cell and then multiplied by the scale factor) and regressed by the percentage of ribosomal gene content, number of features and feature count using the ScaleData Seurat function. The scale factor used was the average gene count per cell. The 2,000 most variable features identified using the variance-stabilizing transformation method or the TF list obtained using the Prowler of Panther 17.0 (ref. ^54^) were used for principal component analysis. The top principal components were used for the Uniform Manifold Approximation and Projection for Dimension Reduction (UMAP) two-dimensional visualization and for graph-based clustering using the FindClusters Seurat function with a resolution of 0.6. The exact number of principal components used was decided based on their s.d. and on random permutation of a subset of data using the JackStraw Seurat function and was generally close to ten principal components. Log-normalized and scaled data were used for violin plots, ball plots and differential expression analysis. Differentially expressed genes in each cluster compared with all clusters were identified using the MAST test implemented in the Seurat function FindMarkers (ref. ^55^). The genes included in the analysis had a log foldchange of at least 0.25 and the Bonferroni adjusted *P* value cutoff was set to *P* < 0.05. The immature MN and V2a clusters (Cluster MN_5_ and V2a_4_) have been excluded from the clustering by TFs. GO analysis of all differentially expressed genes per cluster was performed using Panther (v.17.0) overrepresentation test with Fisher’s exact test. For the regulon analysis, all gene names were converted to mouse gene names using the BioMart Ensembl data mining tool. The analysis was performed on all differentially expressed genes in the TF-derived clusters, using the Cytoscape (v.3.2.0) plugin iRegulon (v.1.3)^56^. Results are ordered by normalized enrichment score. A high normalized enrichment score (>3.0) indicates an RNA binding motif that matches with a large proportion of differentially expressed genes in the analyzed cluster. Subsequently, iRegulon identifies the optimal subset of genes (target genes) that are probably controlled by this motif, and finally associates the motif with the TF (regulator) that is most probably regulating the gene regulatory network associated with that motif. To identify the shared correlation structures between the two datasets in Fig. 6, the package Harmony (v.0.1.0) was used^57^. The larval data used for the analysis in Extended Data Fig. 8 has GEO accession number GSE232801 and the file used was GSE232801_Neurons.RDS (ref. ^45^). The analysis of the authors has been reproduced using the provided script^45^.

### Selective labeling of MNs

The protocol for labeling MNs by injection of dextran dyes (tetramethylrhodamine-dextran, MW 3000, Thermo Fisher, catalog no. D3308; Alexa Fluor 647-dextran, MW 10,000, Thermo Fisher, catalog no. D22914) in the muscles was described in detail in a previously published paper^41^. Briefly, animals were anesthetized with 0.03% tricaine methanesulfonate (MS-222, Sigma-Aldrich, catalog no. E10521) and the tracers were injected using a minutien pin in a selective type of muscle (slow and intermediate or fast). Animals were left to recover for at least 2 h before electrophysiology experiments, immunohistology or in situ hybridization assays.

### Ex vivo adult zebrafish preparation

Electrophysiological recordings were performed using a spinal-cord-brainstem ex vivo preparation of adult zebrafish (8–11 weeks old) of either sex according to previously published protocols^32,58,59^. Animals were deeply anesthetized with 0.03% MS-222 and then dissected in a slush of frozen extracellular solution. The internal organs, axial musculature, skull and vertebral arches were removed. A portion of the axial caudal muscles was left intact to record ventral root motor activity by placing an extracellular recording electrode at an intramyotomal cleft. Most of the brain was also dissected away, leaving only the brainstem region. The preparation was then placed in the recording chamber and perfused with oxygenated extracellular solution at room temperature (20–22 °C) for the duration of the experiment.

### Electrophysiology

A fluorescence microscope (Axioskop FS Plus, Zeiss) equipped with infrared-differential interference contrast optics and a CCD (charge-coupled device) camera with a frame grabber (Hamamatsu) was used to target neurons. Neurons were identified by their expression of fluorescent proteins (GFP and/or RFP) driven by the promoters of different genes (*chx10*, *esrrga*, *shox2*, *vachta*) or by their labeling with fluorescent dyes achieved through muscle injections. All recordings were performed using whole-cell patch-clamp techniques. Glass capillaries were pulled using a micropipette puller (P-1000, Sutter Instruments) from borosilicate glass (Hilgenberg) and were filled with an intracellular solution containing 120 mM K-gluconate, 5 mM KCl, 10 mM HEPES, 4 mM Mg_2_ATP, 0.3 mM Na_4_GTP, 10 mM Na-phosphocreatine, at pH 7.4 adjusted with KOH and an osmolality of 270–280 mOsm to which was added 0.25% neurobiotin (Vector Laboratories, catalog no. SP-1120) to allow for post hoc morphology reconstruction. The meninges were pierced with dedicated glass pipettes using motorized micromanipulators (SM7, Luigs and Neumann), to access the neurons for electrophysiological recordings. Patch-clamp electrodes were driven to the identified neurons while applying constant positive pressure and whole-cell recordings were performed. Intracellular signals were recorded in current clamp with no bias current and were amplified using a MultiClamp 700B amplifier (Molecular Devices) and low pass filtered at 10 kHz. Electrophysiological data were digitized at 10 or 20 kHz using a Digidata 1322A analog-to-digital converter (Molecular Devices) and acquired using pClamp software (v.10; Molecular Devices). Synaptic connectivity between pairs of V2a INs and MNs was examined using dual whole-cell patch-clamp recordings. Action potentials were elicited in presynaptic V2a INs with current injections (3 ms pulses) and monosynaptic excitatory postsynaptic potentials (EPSPs) were recorded in postsynaptic MNs. Fictive swimming was elicited by electrical stimulation of descending axons with an extracellular glass pipette that was placed at the junction between the brainstem and the spinal cord^19,31,32,59^.

### Analysis of electrophysiological data

All MNs and V2a INs included in this study had stable membrane potentials at or below −50 mV, fired action potentials to suprathreshold depolarizations and showed minimal changes in series resistance (<5%). The action potential voltage threshold of V2a INs was determined as the membrane potential at which the dV/dt exceeded 10 V.s^−^^1^. The input resistance was calculated as the slope of the linear part of the current-voltage curve obtained by injection of hyperpolarizing current steps. The minimum recruitment frequency was defined as the slowest swimming frequency at which the neurons fired action potentials during at least two consecutive cycles. The classification of V2a INs into slow, intermediate and fast subtypes took into account the minimum recruitment frequency during fictive locomotion, action potential voltage threshold and membrane resistance. Monosynaptic EPSPs were averaged over 50–200 consecutive sweeps, and their amplitude was calculated as the difference between the baseline and the EPSP peak.

### Immunohistochemistry and in situ hybridization

Spinal cords were dissected after terminal anesthesia with 0.1% MS-222 or after electrophysiological recordings and transferred into 4% paraformaldehyde in PBS (0.01 M; pH 7.4) solution overnight at 4 °C. The tissue was then washed three times for 5 min in PBS. Nonspecific protein binding sites were blocked with 4% normal donkey serum and 1% bovine serum albumin (Sigma-Aldrich, catalog no. A2153) in a solution of 0.5% Triton X-100 (Sigma-Aldrich, catalog no. T9284) in PBS for 30 min at room temperature. Spinal cords were incubated with anti-GFP (chicken polyclonal, Abcam, catalog no. ab13970, 1:1,000) and anti-mCherry (rabbit polyclonal, Abcam, catalog no. ab167453, 1:1,000). After thorough buffer rinses, the tissue was then incubated overnight at 4 °C with the appropriate Alexa Fluor-conjugated secondary antibody anti-chicken 488 (Thermo Fisher, catalog no. A11039) or anti-rabbit 568 (Thermo Fisher, catalog no. A10042) 1:1,000 in 0.5% Triton X-100 in PBS. To label neurobiotin-filled neurons, streptavidin conjugated to Alexa Fluor 647 (Thermo Fisher, catalog no. S32357) 1:1,000 in 0.5% Triton X-100 in PBS was added. The spinal cords were then rinsed thoroughly in PBS and mounted in 80% glycerol in PBS.

For RNAscope analyses, custom probes were designed by Advanced Cell Diagnostics (Dr-calb1, Dr-chrna2b, Dr-grin1b, Dr-hoxb13a, Dr-neurod1, Dr-pvalb6, Dr-esrrga, Dr-shox2, Dr-vachta). Spinal cords from zebrafish (7–8 weeks old) were dissected out and fixed with 4% paraformaldehyde in PBS for 24 h at 4 °C. The RNAscope Multiplex Fluorescent v.2 Assay (Advanced Cell Diagnostics) was performed according to the manufacturer’s instructions with a few modifications. In brief, spinal cords were washed in PBS for 30 min, incubated in Target Retrieval Buffer (heated to boiling point) for 3 min, washed for 2 min with MilliQ water and dehydrated for 3 min in 100% ethanol at room temperature. The following incubations were then performed in a thermoblock at 40 °C, with wash steps in between of two times 1 min in washing buffer on a shaker: Protease III (10 m), Probe in C1 (Calb1, Chrna2b, Grin1b, Hoxb13a, Esrrga), and/or C2 (NeuroD1, Shox2) and/or C3 (Pvalb6, vAChTa), depending on the probe combination (2 h), AMP 1 (30 min), AMP 2 (30 min) and AMP 3 (15 min). If C1 probe was present: horseradish peroxidase (HRP)-C1 (15 min), tyramide signal amplification (TSA) (30 min), HRP blocker (15 min). If C2 probe was present: HRP-C2 (15 min), TSA (30 min), HRP blocker (15 min). If C3 probe was present: HRP-C3 (15 min), TSA (30 min), HRP blocker (15 min). TSA dyes used were TSA Plus Cyanine 5 (Cy5, Akoya Biosciences, catalog no. NEL745001KT) and TSA Plus Cyanine 3 (Cy3, Akoya Biosciences, catalog no. NEL744001KT). If required, the RNAscope protocol was followed by immunohistochemistry.

### Image analysis and morphology reconstruction

A laser scanning confocal microscope (Zeiss LSM 980-Airy) was used to acquire whole-mount imaging of the spinal cords with a ×20 or ×40 water objective. For RNAscope analysis, spinal cords were positioned laterally on a microscope glass slide and then mounted with ProLong Gold Antifade Mountant (Thermo Fisher, catalog no. P36930). The soma size was measured as the maximum surface area from confocal images. The position of axonal projections was measured from confocal images of two spinal segments away from the soma. The full morphologies (soma, axons and dendrites) of neurobiotin-filled neurons were traced and reconstructed manually in Adobe Illustrator (Adobe Systems Inc.) on *z*-stacks of confocal images. For RNAscope analysis, only intact spinal cords in the proper orientation were used. RNAscope puncta were counted manually on *z*-stacks of confocal images and neurons were classified as positive for the RNA probe if they had at least three puncta at the level of the cell body. Double positive neurons for RNAscope probes were identified if they show at least three puncta of each probe. MNs were identified based on the selective muscle retrogradely injected and their respective positions in the spinal cord. Images were analyzed using ImageJ and the top, bottom, lateral and medial (central canal) coordinates of the spinal cord were used as references to calculate the position of the cell soma. Graphs were generated with Prism (v.7 and 9) or using Seaborn, a Python data visualization library based on matplotlib54. Figures were prepared with Corel Draw (Corel Corporation), Adobe Photoshop and Adobe Illustrator.

### Ablation and behavioral tests

The transgenic line Tg(*esrrga*-hs:loxP-RFP-loxP-GFP) crossed with Tg(*chx10*:GFP) was used for selective ablation of Esrrga^+^ V2a INs and the transgenic line Tg(*vachta*:GFP) crossed with Tg(*chx10*:RFP) was used for selective ablation of vAChTa^+^ V2a INs. Zebrafish (6 weeks old) were anesthetized and embedded in 1.5% low-melt agarose in a Petri dish. The gills and mouth were exposed from the agarose and the Petri dish was filled with fish water containing 0.01% MS-222. They were then placed under a two-photon/confocal microscope (Zeiss LSM 980-Airy) and Esrrga^+^ or vAChTa^+^ V2a INs were photoablated (wavelength 800 nm). Ablation was performed bilaterally over approximately 17 segments (150 Esrrga^+^ or 80 vAChTa^+^ V2a INs ablated). The cholinergic esV2a INs were not ablated in these experiments. Control animals were embedded alongside the ablated zebrafish but were not subjected to two-photon laser ablation. Successful ablations were confirmed by the permanent loss of GFP fluorescence. Both the ablated and control zebrafish were allowed to recover from anesthesia for at least 1 h at 28 °C before behavioral analysis.

For behavioral analysis, animals were placed in a circular dish containing fish water positioned on a plexiglass platform, illuminated from below by a light-emitting diode lightbox and imaged from above with a high-speed camera. Control and ablated fish were tested in randomized order. Fish were placed in an 8-cm-diameter circular glass dish filled with 25 ml of fish water and were allowed to acclimate for 20 min. Swimming was induced by a tactile stimulus applied to the tail using a fine tungsten pin and was recorded at 350 frames per second. Escape was evoked by a brief sound stimulus (10 ms, sine wave at 500 Hz) delivered by an audio speaker that was fixed on the plexiglass platform. Evoked escape was recorded at 350 frames per second. Trials in which stimulation failed to elicit a C-start escape maneuver were excluded from the analysis.

### Behavioral data analysis

For each experiment, videos were analyzed using DeepLabCut (v.2.2.06). The full skeleton of the fish was tracked and the instantaneous velocity, distance traveled and bend amplitudes were analyzed using a custom MATLAB script (10.5281/zenodo.7252046)^2^ with distance and velocity values expressed as body lengths (bl) and body lengths per second (bl s^−^^1^), respectively. Data are presented as averages of three to four recordings per animal.

### Quantification and statistical analysis

Statistical analysis for electrophysiological and anatomical experiments was performed in Prism (v.7 and v.9) and all data were tested for normality. Statistical analysis for transcriptomic data was performed in R as described in the transcriptomic analysis section. Differential expression analysis is based on nonparametric Wilcoxon rank sum test. No adjustments were made for multiple comparisons. Gene expression in Figs. 2m and 5p was normalized to the neuron with maximum gene expression level for each gene. The number of puncta in Fig. 2n was normalized to the neuron with maximum puncta for each probe. Data were tested for normality and two-tailed Student’s *t*-test or Mann–Whitney *U* test was used for two groups while one-way analysis of variance (ANOVA) with Tukey’s post hoc multiple comparisons or Kruskal–Wallis with Dunn’s multiple comparisons tests were used for more than two groups, one condition, as appropriate. Data are reported as violin, box and whisker plots or as mean ± s.e.m. with corresponding statistical tests and *n* numbers in figure legends. Results were considered statistically significant if *P* < 0.05 (*P ≤ 0.05, **P ≤ 0.01, ***P ≤ 0.001, ****P ≤ 0.0001) and for transcriptomic data if Bonferroni adjusted *P* < 0.05.

### Statistics and reproducibility

The experiments shown in Fig. 2c,d,i,j were repeated independently in four animals for each RNAscope probe combination, with similar results. The experiments shown in Fig. 3a were repeated independently in eight animals, with similar results. The experiments shown in Fig. 3e were repeated independently in three animals, with similar results. The experiments shown in Fig. 5b,e,h were repeated independently in four animals for each transgenic line, with similar results. The experiments shown in Extended Data Fig. 4a,c,e,g were repeated independently in four animals for each RNAscope probe combination, with similar results. No statistical methods were used to predetermine sample sizes, but our sample sizes are similar to those reported in our previous publications^18,23,24,60^ and based on the 3R principle. All samples were allocated randomly into experimental groups. The investigators were blinded to group allocation during data analysis. During data collection, blinding was not relevant given the methodological approach (scRNA-seq sample preparation, electrophysiological recording) or the experimental design (ablation and behavioral test).

### Reporting summary

Further information on research design is available in the Nature Portfolio Reporting Summary linked to this article.

## Online content

Any methods, additional references, Nature Portfolio reporting summaries, source data, extended data, supplementary information, acknowledgements, peer review information; details of author contributions and competing interests; and statements of data and code availability are available at 10.1038/s41593-023-01479-1.

### Supplementary information

Reporting Summary

### Source data

Source Data Fig. 2 Statistical source data for Fig. 2e,f,k–m,o,p.

Source Data Fig. 3 Statistical source data for Fig. 3c,d,g,i.

Source Data Fig. 5 Statistical source data for Fig. 5j–q.

Source Data Fig. 7 Statistical source data for Fig. 7c–f.

Source Data Extended Data Fig. 1 Statistical source data for Fig. 1b,c.

Source Data Extended Data Fig. 3 Statistical source data for Fig. 3c.

Source Data Extended Data Fig. 4 Statistical source data for Fig 4b,d,f,h,i.

Source Data Extended Data Fig. 7 Statistical source data for Fig. 7d,f.

## Data Availability

The transcriptomic dataset generated in this study has been deposited in the GEO database under accession code GSE243993. The larval transcriptomic dataset analyzed in Extended Data Fig. 8 is available under accession code GSE232801. Source data are provided with this paper. Other data are available from the corresponding authors upon reasonable request.

## Code availability

All custom codes written for this study are available at https://github.com/ElManiraLab/sc-RNAseq/ and 10.5281/zenodo.7252046. No custom software codes were used for figures produced in Python.
