# Supplementary material for: Molecular blueprints for spinal circuit modules controlling locomotor speed in zebrafish
Source: Nat Neurosci. 2023 Nov 2;27(1):78–89. doi: 10.1038/s41593-023-01479-1 (PMC10774144; doi:10.1038/s41593-023-01479-1)
Supplement: Supplementary file 1 — Reporting Summary [file 41593_2023_1479_MOESM1_ESM.pdf]

nature portfolio

Corresponding author(s): El Manira

Last updated by author(s): Sep 29, 2023

Reporting Summary

Nature Portfolio wishes to improve the reproducibility of the work that we publish. This form provides structure for consistency and transparency in reporting. For further information on Nature Portfolio policies, see our Editorial Policies and the Editorial Policy Checklist.

Statistics

For all statistical analyses, confirm that the following items are present in the figure legend, table legend, main text, or Methods section.

n/a	Confirmed	
☐	☒	The exact sample size (n) for each experimental group/condition, given as a discrete number and unit of measurement
☐	☒	A statement on whether measurements were taken from distinct samples or whether the same sample was measured repeatedly
☐	☒	The statistical test(s) used AND whether they are one- or two-sided<br>*Only common tests should be described solely by name; describe more complex techniques in the Methods section.*
☒	☐	A description of all covariates tested
☐	☒	A description of any assumptions or corrections, such as tests of normality and adjustment for multiple comparisons
☐	☒	A full description of the statistical parameters including central tendency (e.g. means) or other basic estimates (e.g. regression coefficient) AND variation (e.g. standard deviation) or associated estimates of uncertainty (e.g. confidence intervals)
☐	☒	For null hypothesis testing, the test statistic (e.g. F, t, r) with confidence intervals, effect sizes, degrees of freedom and P value noted<br>*Give P values as exact values whenever suitable.*
☒	☐	For Bayesian analysis, information on the choice of priors and Markov chain Monte Carlo settings
☒	☐	For hierarchical and complex designs, identification of the appropriate level for tests and full reporting of outcomes
☒	☐	Estimates of effect sizes (e.g. Cohen's d, Pearson's r), indicating how they were calculated

Our web collection on statistics for biologists contains articles on many of the points above.

Software and code

Policy information about availability of computer code

Data collection	Multiclamp and pClamp (version 10, Molecular Devices) was used for electrophysiological data collection. Zeiss LSM 980-Airy confocal microscope was used for morphological and anatomical data collection.
Data analysis	Electrophysiological data was analyzed using pClamp (version 10, Molecular Devices). Anatomical images were analyzed using ImageJ (ImageJ version 1.53t). Graphs were generated using Prism (versions 7 and 9; GraphPad Software). Transcriptomic data was analyzed using R. STAR (version 2.5.3a), R (version 4.0.5, R core team, 2022), Seurat package (version 4.0.2), DeepLabCut (version 2.2.06), Panther (version 17.0), Cytoscape (version 3.2.0) plugin iRegulon (version 1.3), Harmony (version 0.1.0).<br>All custom codes written for this study are available at https://github.com/ElManiraLab/sc-RNAseq/. No custom software codes were used for figures produced in Python.

For manuscripts utilizing custom algorithms or software that are central to the research but not yet described in published literature, software must be made available to editors and reviewers. We strongly encourage code deposition in a community repository (e.g. GitHub). See the Nature Portfolio guidelines for submitting code & software for further information.

Data

Policy information about availability of data

All manuscripts must include a data availability statement. This statement should provide the following information, where applicable:

- Accession codes, unique identifiers, or web links for publicly available datasets
- A description of any restrictions on data availability
- For clinical datasets or third party data, please ensure that the statement adheres to our policy

The reads from each sequenced cell were mapped to the zebrafish reference genome "Danio_rerio, Ensembl, GRCz11". The transcriptomic dataset generated in this study has been deposited in the GEO database under accession code GSE243993. The larval transcriptomic dataset analyzed in Extended Data Fig. 8 is available under accession code GSE232801. Source Data for figures produced in Python are available as Supplementary Information. Other data are available from the corresponding authors upon reasonable request.

Human research participants

Policy information about studies involving human research participants and Sex and Gender in Research.

Reporting on sex and gender	N/A
Population characteristics	N/A
Recruitment	N/A
Ethics oversight	N/A

Note that full information on the approval of the study protocol must also be provided in the manuscript.

Field-specific reporting

Please select the one below that is the best fit for your research. If you are not sure, read the appropriate sections before making your selection.

☒ Life sciences ☐ Behavioural & social sciences ☐ Ecological, evolutionary & environmental sciences

For a reference copy of the document with all sections, see nature.com/documents/nr-reporting-summary-flat.pdf

Life sciences study design

All studies must disclose on these points even when the disclosure is negative.

Sample size	No statistical methods were used to pre-determine sample sizes, but our sample sizes are similar to those reported in our previous publications (refs 17,22,23,59 in the paper) and based on the 3R principle.
Data exclusions	In the transcriptomics dataset, we excluded genes that were expressed in less than 3 cells, and cells that had more than 30% reads from mitochondrial genes or from spike-ins, or a number of genes higher than 9000 or lower than 2000.
Replication	The experiments shown in Fig. 2c,d,i,j were repeated independently in 4 animals for each RNAscope probe combination, with similar results. The experiments shown in Fig. 3a were repeated independently in 8 animals, with similar results. The experiments shown in Fig. 3e were repeated independently in 3 animals, with similar results. The experiments shown in Fig. 5b,e,h were repeated independently in 4 animals for each transgenic line, with similar results. The experiments shown in Extended Data Fig. 4a, 4c, 4e, 4g were repeated independently in 4 animals for each RNAscope probe combination, with similar results.
Randomization	In ablation experiments, control and ablated fish were tested in randomized order. All samples were randomly allocated into experimental groups.
Blinding	The investigators were blinded to group allocation during data analysis. During data collection blinding was not relevant given the methodological approach (single-cell RNA sequencing sample preparation, electrophysiological recording) or the experimental design (ablation and behavioral test).

Reporting for specific materials, systems and methods

We require information from authors about some types of materials, experimental systems and methods used in many studies. Here, indicate whether each material, system or method listed is relevant to your study. If you are not sure if a list item applies to your research, read the appropriate section before selecting a response.

Materials & experimental systems

n/a	Involved in the study	
☐	☒	Antibodies
☒	☐	Eukaryotic cell lines
☒	☐	Palaeontology and archaeology
☐	☒	Animals and other organisms
☒	☐	Clinical data
☒	☐	Dual use research of concern

Methods

n/a	Involved in the study	
☒	☐	ChIP-seq
☒	☐	Flow cytometry
☒	☐	MRI-based neuroimaging

Antibodies

Antibodies used

1. Anti-GFP IgY, chicken polyclonal, Abcam, Cat# ab13970, LOT# GR3361051-10, RRID:AB_300798
2. Anti-mCherry IgG, rabbit polyclonal, Abcam, Cat# ab167453, LOT# GR3358274-1, RRID:AB_2571870
3. Goat anti-chicken IgY (H+L) polyclonal secondary, Alexa Fluor™ 488-conjugated, Thermo Fisher Scientific, Cat# A-11039, LOT# 2420700, RRID:AB_2534096
4. Donkey anti-rabbit IgG (H+L) highly cross-adsorbed polyclonal secondary, Alexa Fluor™ 568-conjugated, Thermo Fisher Scientific, Cat# A10042, LOT# 1668655, RRID:AB_2534017

Validation

The antibodies listed can be found on these commercially available websites. Validation and relevant citations are provided on the supplier's website.
1. https://www.abcam.com/en-se/products/primary-antibodies/anti-gfp-antibody-ab13970
2. https://www.abcam.com/en-se/products/primary-antibodies/anti-mcherry-antibody-ab167453
3. https://www.thermofisher.com/antibody/product/Goat-anti-Chicken-IgY-H-L-Secondary-Antibody-Polyclonal/A-11039
4. https://www.thermofisher.com/antibody/product/Donkey-anti-Rabbit-IgG-H-L-Highly-Cross-Adsorbed-Secondary-Antibody-Polyclonal/A10042

Animals and other research organisms

Policy information about studies involving animals; ARRIVE guidelines recommended for reporting animal research, and Sex and Gender in Research

Laboratory animals	Zebrafish, Danio rerio (ablation: 6 week old, electrophysiology: 8 to 11 week old, sequencing: 7 week old, TNAscope: 7-8 week old)
Wild animals	no wild animals were used
Reporting on sex	both sexes were used
Field-collected samples	No filed collect samples were used
Ethics oversight	All experimental procedures followed the EU guidelines and were approved by the Animal Research Ethical Committee in Stockholm (Stockholms djurförsöksetiska nämnd, Dnr 6517-2019 and Dnr 19429-2022).

Note that full information on the approval of the study protocol must also be provided in the manuscript.
